# 2020 Ebola virus disease outbreak in Équateur Province, Democratic Republic of the Congo: a retrospective genomic characterisation

**DOI:** 10.1016/S2666-5247(23)00259-8

**Published:** 2024-02

**Authors:** Eddy Kinganda-Lusamaki, Shannon Whitmer, Emmanuel Lokilo-Lofiko, Adrienne Amuri-Aziza, Francisca Muyembe-Mawete, Jean Claude Makangara-Cigolo, Gerry Makaya, Francis Mbuyi, Amy Whitesell, Ruth Kallay, Mary Choi, Catherine Pratt, Daniel Mukadi-Bamuleka, Hugo Kavunga-Membo, Meris Matondo-Kuamfumu, Fabrice Mambu-Mbika, Richard Ekila-Ifinji, Trevor Shoemaker, Miles Stewart, Julia Eng, Abraham Rajan, Gnakub N Soke, Peter N Fonjungo, John Otokoye Otshudiema, Gervais Léon Tengomo Folefack, Elisabeth Pukuta-Simbu, Emir Talundzic, Elizabeth Shedroff, Jacques Likofata Bokete, Anaïs Legand, Pierre Formenty, Christopher N Mores, Abigail J Porzucek, Sarah R Tritsch, John Kombe, Gaston Tshapenda, Felix Mulangu, Ahidjo Ayouba, Eric Delaporte, Martine Peeters, Michael R Wiley, Joel M Montgomery, John D Klena, Jean-Jacques Muyembe-Tamfum, Steve Ahuka-Mundeke, Placide Mbala-Kingebeni

**Affiliations:** a Pathogen Genomics Laboratory, Institut National de Recherche Biomédicale, Kinshasa, Democratic Republic of the Congo; b Service de Microbiologie, Cliniques Universitaires, Faculté de Médecine, Université de Kinshasa, Kinshasa, Democratic Republic of the Congo; c Viral Special Pathogens Branch, US Centers for Disease Control and Prevention, Atlanta, GA, USA; d Emergency Response and Recovery Branch USA, US Centers for Disease Control and Prevention, Atlanta, GA, USA; e Vysnova Partners, Landover, MD, USA; f Department of Environmental, Agricultural and Occupational Health, College of Public Health, University of Nebraska Medical Center, Omaha, NE, USA; g PraesensBio, Omaha, NE, USA; h WHO Health Emergencies Programme, WHO, Kinshasa, Democratic Republic of the Congo; i Global Health Department, Milken Institute School of Public Health, The George Washington University, Washington, DC, USA; j Ministry of Health, Kinshasa, Democratic Republic of the Congo; k TransVIHMI, University of Montpellier, Institut de Recherche pour le Développement, INSERM, Montpellier, France; l Johns Hopkins University Applied Physics Laboratory, Johns Hopkins University, Laurel, MD, USA; m Health Emergencies Programme, WHO, Geneva, Switzerland; n Division of Global Health Protection, US Centers for Disease Control and Prevention, Kinshasa, Democratic Republic of the Congo; o Division of Global HIV and Tuberculosis, US Centers for Disease Control and Prevention, Kinshasa, Democratic Republic of the Congo

## Abstract

**Background:**

The Democratic Republic of the Congo has had 15 Ebola virus disease (EVD) outbreaks, from 1976 to 2023. On June 1, 2020, the Democratic Republic of the Congo declared an outbreak of EVD in the western Équateur Province (11th outbreak), proximal to the 2018 Tumba and Bikoro outbreak and concurrent with an outbreak in the eastern Nord Kivu Province. In this Article, we assessed whether the 11th outbreak was genetically related to previous or concurrent EVD outbreaks and connected available epidemiological and genetic data to identify sources of possible zoonotic spillover, uncover additional unreported cases of nosocomial transmission, and provide a deeper investigation into the 11th outbreak.

**Methods:**

We analysed epidemiological factors from the 11th EVD outbreak to identify patient characteristics, epidemiological links, and transmission modes to explore virus spread through space, time, and age groups in the Équateur Province, Democratic Republic of the Congo. Trained field investigators and health professionals recorded data on suspected, probable, and confirmed cases, including demographic characteristics, possible exposures, symptom onset and signs and symptoms, and potentially exposed contacts. We used blood samples from individuals who were live suspected cases and oral swabs from individuals who were deceased to diagnose EVD. We applied whole-genome sequencing of 87 available Ebola virus genomes (from 130 individuals with EVD between May 19 and Sept 16, 2020), phylogenetic divergence versus time, and Bayesian reconstruction of phylogenetic trees to calculate viral substitution rates and study viral evolution. We linked the available epidemiological and genetic datasets to conduct a genomic and epidemiological study of the 11th EVD outbreak.

**Findings:**

Between May 19 and Sept 16, 2020, 130 EVD (119 confirmed and 11 probable) cases were reported across 13 Équateur Province health zones. The individual identified as the index case reported frequent consumption of bat meat, suggesting the outbreak started due to zoonotic spillover. Sequencing revealed two circulating Ebola virus variants associated with this outbreak—a Mbandaka variant associated with the majority (97%) of cases and a Tumba-like variant with similarity to the ninth EVD outbreak in 2018. The Tumba-like variant exhibited a reduced substitution rate, suggesting transmission from a previous survivor of EVD.

**Interpretation:**

Integrating genetic and epidemiological data allowed for investigative fact-checking and verified patient-reported sources of possible zoonotic spillover. These results demonstrate that rapid genetic sequencing combined with epidemiological data can inform responders of the mechanisms of viral spread, uncover novel transmission modes, and provide a deeper understanding of the outbreak, which is ultimately needed for infection prevention and control during outbreaks.

**Funding:**

WHO and US Centers for Disease Control and Prevention.

## Introduction

**Figure 1:**
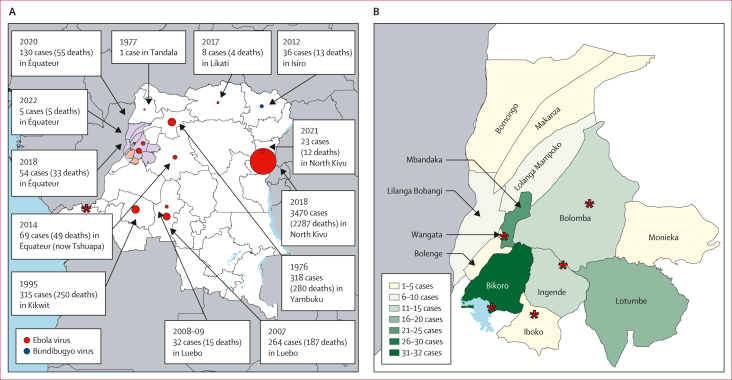
Ebola virus outbreaks in the Democratic Republic of the Congo, 1976–2022 (A) The distribution of Ebola virus disease outbreaks in the Democratic Republic of the Congo. Coloured circles identify locations of previous outbreaks and the size of circles represents the number of positive human cases. The map shows the affected 2020 health zones (orange and purple shading) and sites of the 2018 Équateur outbreak (orange shading). The red star indicates the location of the Kinshasa diagnostic laboratory during the Équateur Province 2020 outbreak. (B) The locations and prevalence of Ebola virus disease cases during the 2020 Ebola virus disease outbreak in the Équateur Province. The red stars indicate the location of the diagnostic and field laboratories during the Équateur Province 2020 outbreak (Mbandaka, Ingende, Itipo, Bikoro, and Bolomba).

On June 1, 2020, the Democratic Republic of the Congo declared an outbreak of Ebola virus disease (EVD) in Mbandaka, the capital of Équateur Province.^1^ This declaration represented the 11th known EVD outbreak in the Democratic Republic of the Congo and occurred close to the location of the 2018 Équateur Province outbreak in the Bikoro health zone (figure 1A).^2^ From Sept 1, 1976, to Aug 22, 2022, the Democratic Republic of the Congo has experienced 15 EVD outbreaks. Four additional EVD outbreaks have occurred in the Democratic Republic of the Congo since the 11th Équateur Province outbreak (figure 1A).^3^ Historically, most EVD outbreaks have occurred in the northern half of the Democratic Republic of the Congo and have been mainly caused by Ebola virus (EBOV; figure 1A), although Bundibugyo virus caused an outbreak in Isiro, Haut-Uélé Province (formerly, Orientale Province) in 2012.^4^

**Research in context**

**Evidence before this study**

From Sept 1, 1976, to Aug 22, 2022, the Democratic Republic of the Congo has had 15 Ebola virus disease (EVD) outbreaks. These EVD outbreaks have been distributed across the north of the Democratic Republic of the Congo and have been mostly caused by Ebola virus (EBOV), although Bundibugyo virus has caused a single outbreak. We searched PubMed for all studies examining previous filovirus outbreaks published between Jan 1, 1999, and March 1, 2023, using the search terms “Ebolavirus outbreak” and “Ebolavirus outbreak DRC”. We did not apply language restrictions. We found that multiple studies had examined either the epidemiological, clinical, or genetic components of previous outbreaks, but few studies combined both the epidemiological and genetic data into a broad picture of an EVD outbreak.

**Added value of this study**

In this study, we review the epidemiological and genetic details associated with the 2020 EVD outbreak in Équateur Province (the 11th outbreak). The 11th outbreak occurred concurrently with an ongoing EVD outbreak in the eastern Nord Kivu Province. Between May 19 and Sept 16, 2020, 130 EVD cases (119 confirmed and 11 probable) were reported across 13 Équateur Province health zones. The index case reported frequent consumption of bat meat, suggesting the outbreak started because of zoonotic spillover and was not related to the ongoing EVD outbreak in the eastern Nord Kivu Province. Sequencing revealed two circulating EBOV variants associated with this outbreak—a Mbandaka variant associated with 84 (97%) of 87 human cases and a Tumba-like variant from four cases, with similarity to the 2018 EBOV sequences from the ninth EVD outbreak. The Tumba-like variant exhibited a reduced substitution rate, suggesting transmission from a previous EVD survivor. Integrating genetic sequencing and epidemiological data allowed for investigative fact-checking and uncovered additional unreported cases of nosocomial transmission.

**Implications of all the available evidence**

The data presented in this Article demonstrate co-circulation of two EBOV variants consistent with two separate concurrent outbreaks. The first outbreak was initiated as a result of zoonotic transmission. Concurrent with the first outbreak, a second outbreak started due to transmission from an EVD survivor who was persistently infected from the Tumba 2018 outbreak. These data illustrate the importance of next-generation sequencing as an epidemiological tool to identify and connect chains of transmission.

Ebolavirus species have not been definitively identified in bat hosts; however, Amman and colleagues^5^ have directly identified, isolated, and sequenced Marburg virus, another filovirus, from captured bats, supporting the hypothesis that new filovirus infections can arise from an animal reservoir. Bats seropositive for Ebola virus were also identified in the Nord Kivu Province and Équateur Province regions of the Democratic Republic of the Congo and in the neighbouring Republic of the Congo during the EVD outbreak in 2018.^6^^,^^7^ In previous EVD outbreaks in Gabon and the Republic of the Congo, multiple virus infections had occurred after handling EBOV-positive gorillas or chimpanzees, and evidence of abrupt ape population declines had also occurred concurrently with human infections.^8^ Furthermore, filovirus persistence in EVD and Marburg virus disease survivors has resulted in new infections through sexual transmission or contact with bodily fluids.^9^^,^^10^ Infectious EBOV has been isolated directly from semen^11^ and vitreous humour,^12^ and viral RNA has been detected in cerebrospinal fluid,^13^ vaginal secretions,^14^ breastmilk, and other bodily fluids^15^ in survivors of EVD. Before the development of rapid genetic sequencing, identifying whether a new EVD outbreak is the result of a zoonotic spillover or due to EVD survivor-related transmission had been challenging. However, recent data demonstrate that strains that have been transmitted from an EVD survivor have a reduced substitution rate.^11^^,^^13^^,^^16^^,^^17^

In this study, we assess the epidemiological and genetic details associated with the 2020 EVD outbreak in Équateur Province. This outbreak occurred in the northwest of the Democratic Republic of the Congo concurrently with an EVD outbreak taking place in the northeast of the Democratic Republic of the Congo in Nord Kivu, Ituri, and Sud Kivu provinces (tenth outbreak), and led to initial suspicion that an individual infected with EBOV transmitted EVD across the Democratic Republic of the Congo.^18^ However, genetic sequencing refuted this hypothesis and demonstrated that the Équateur Province EVD outbreak was independent of the Nord Kivu Province EVD outbreak and that two different EBOV variants were circulating in Équateur Province. Constructing a wide view of the Équateur Province 2020 EVD outbreak in this study, by linking the available epidemiological and genetic data, allowed for investigative fact-checking, verified patient-reported sources of possible zoonotic spillover, uncovered additional unreported cases of nosocomial transmission, and provided a deeper investigation into the 11th outbreak.

## Methods

### Case investigation and contact tracing

We used the Democratic Republic of the Congo Ministry of Health’s EVD case definition to classify cases as suspected, probable, or confirmed (appendix pp 1–3). For suspected cases, the EVD case definition was different to the one used in previous outbreaks in that fever was not a required part of the criteria. This definition was updated because fever is not uniformly present in all confirmed cases of EVD at the time of detection. All suspected, probable, and confirmed cases were investigated to record demographic characteristics, determine possible exposures, document information about symptom onset and signs and symptoms, and identify potentially exposed contacts. Data were collected by trained field investigators and health professionals using a standardised case investigation form (appendix pp 4–6) that was subsequently entered into an electronic database. Additional details are available in the appendix (pp 7–10).

Blood samples from individuals who were live suspected cases and oral swabs from individuals who were deceased suspected cases were collected as part of the Democratic Republic of the Congo Ministry of Health public health emergency response; therefore, consent for sample collection was waived. Ethical approval for this manuscript was provided by the Comité d’Ethique at the Ecole de Sante Publique at the Université de Kinshasa.

### Sample processing, shipment, and sequencing

Confirmation of cases required detection of EBOV RNA in blood or body fluids by RT-PCR. In Kinshasa, Mbandaka, and four other field laboratories (Ingende, Itipo, Bikoro, and Bolomba), specimens were inactivated and tested by RT-PCR (Xpert Ebola Assay, Cepheid, Sunnyvale, CA, USA) to detect EBOV RNA. For sequence analysis, samples were handled in a glovebox and RNA was extracted from the diagnostic specimen using Viral RNA Mini kits (Qiagen, Hilden, Germany). Viral sequencing was done using either amplicon-based MinIon sequencing (Oxford Nanopore Technologies, Oxford, UK) or hybridisation enrichment using EBOV-specific oligonucleotides on the MiSeq or iSeq100 (Illumina, San Diego, CA, USA) platform. Amplicon-based MinIon sequencing was done using EBOV-specific primers designed against the Équateur Province 2020 viral sequence protocol (appendix p 11) and the ARTIC protocol.^19^

### Phylogenetic and Bayesian analysis

We constructed consensus genomes using the bioinformatics method appropriate to the library construction method, either the ARTIC EBOV bioinformatics protocol (for amplicon-based MinIon sequencing),^19^ or a read mapping to a reference genome using in-house scripts (for TruSeq-based Illumina sequencing). Additional details are available in the appendix (pp 7–10).

Viral genomes were aligned using MAFFT (7.450) and maximum likelihood trees were constructed using RAxML (7.3.0-PTHREAD) with 1000 bootstrap iterations. Evolutionary rates were estimated using both linear regression modelling and time-scaled phylogenies. We did linear regression modelling using TempEst (1.5.3) with maximum likelihood trees rooted on the earliest available Mbandaka 2020 or Tumba 2018 sequences (OR084846–932). Genetic distance versus collection date was visualised in R (4.1.3). We calculated evolutionary rates estimates, *R*^*2*^, and p values in R (4.1.3) using lm(distance∼date) with 95% prediction intervals estimated from the same dataset. We did Bayesian analysis using BEAST (1.10.4) with separate Mbandaka 2020 (including closely related sequences from 2014 and 2018 Democratic Republic of the Congo outbreaks) or Tumba 2018 sequence sets. Statistically strong differences in Bayes factor values for different models were not observed (appendix p 12) and we used the uncorrelated log normal skygrid model to maintain uniformity with previous EBOV rate estimates. Additional details are available in the appendix (pp 7–10).

### Quantification and statistical analysis

We did geographical analyses using QGIS (3.16.13) and made choropleth maps using R (4.1.2). Analysis of epidemiological metadata was done using Python (3.9.13) in Jupyter Notebook (6.4.12). The Democratic Republic of the Congo population demographic data were accessed from the WHO Demographic Yearbook, 2020.^20^ Maximum clade credibility time-scaled phylogenies were visualised using ggtree (3.2.0) and root-to-tip versus specimen collection dates were visualised using R (4.1.3) in Jupyter Notebook (6.4.12). Epidemiological and genetic data were visualised in ChainChecker (2.0).

### Role of the funding source

The funders of the study had no role in study design, data collection, data analysis, data interpretation, or writing of the report.

## Results

**Figure 2:**
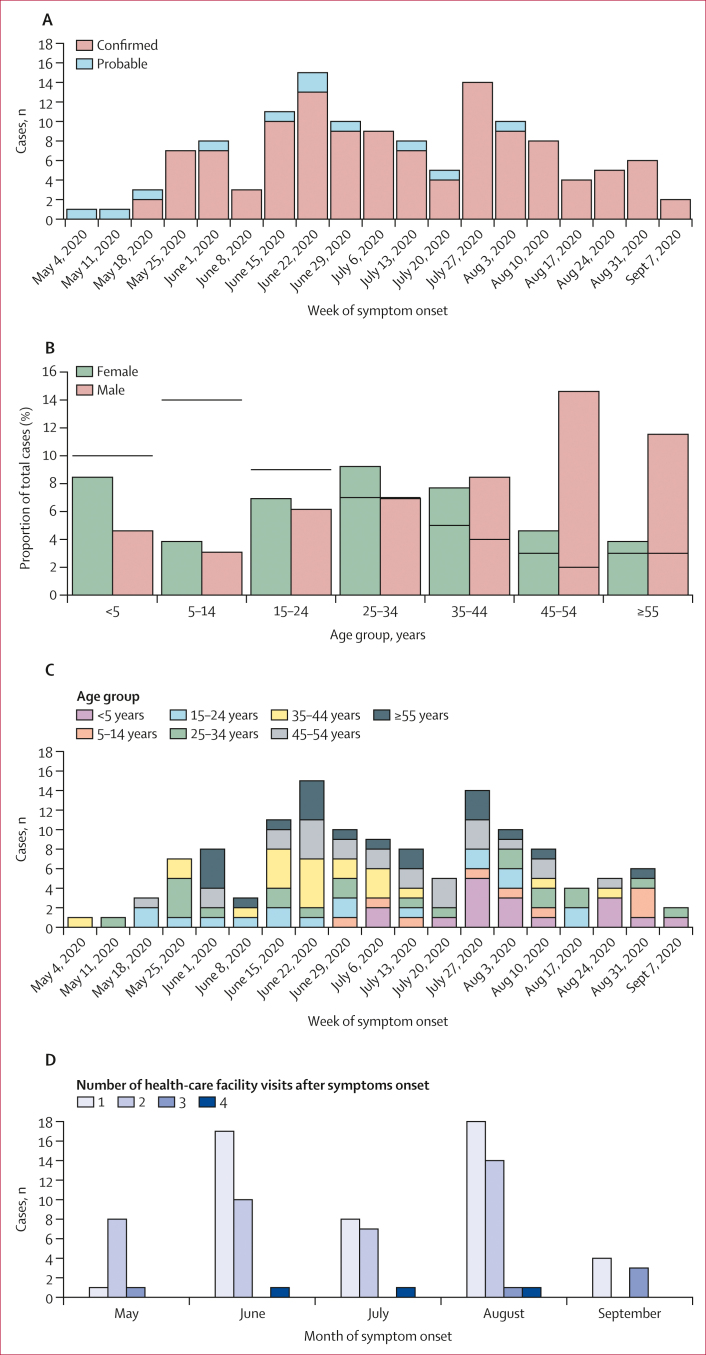
Demographics of EVD cases during the 2020 Équateur Province outbreak (A) Epidemiological curve of confirmed and probable EVD cases over time. (B) Age distribution of confirmed and probable EVD cases by gender (the black horizontal bars represent the 2020 Democratic Republic of the Congo known age and gender population distribution from WHO). (C) Temporal age distribution of individuals with EVD. (D) Distribution of patients with EVD who visited multiple health-care facilities after symptoms onset. EVD=Ebola virus disease.

**Figure 3:**
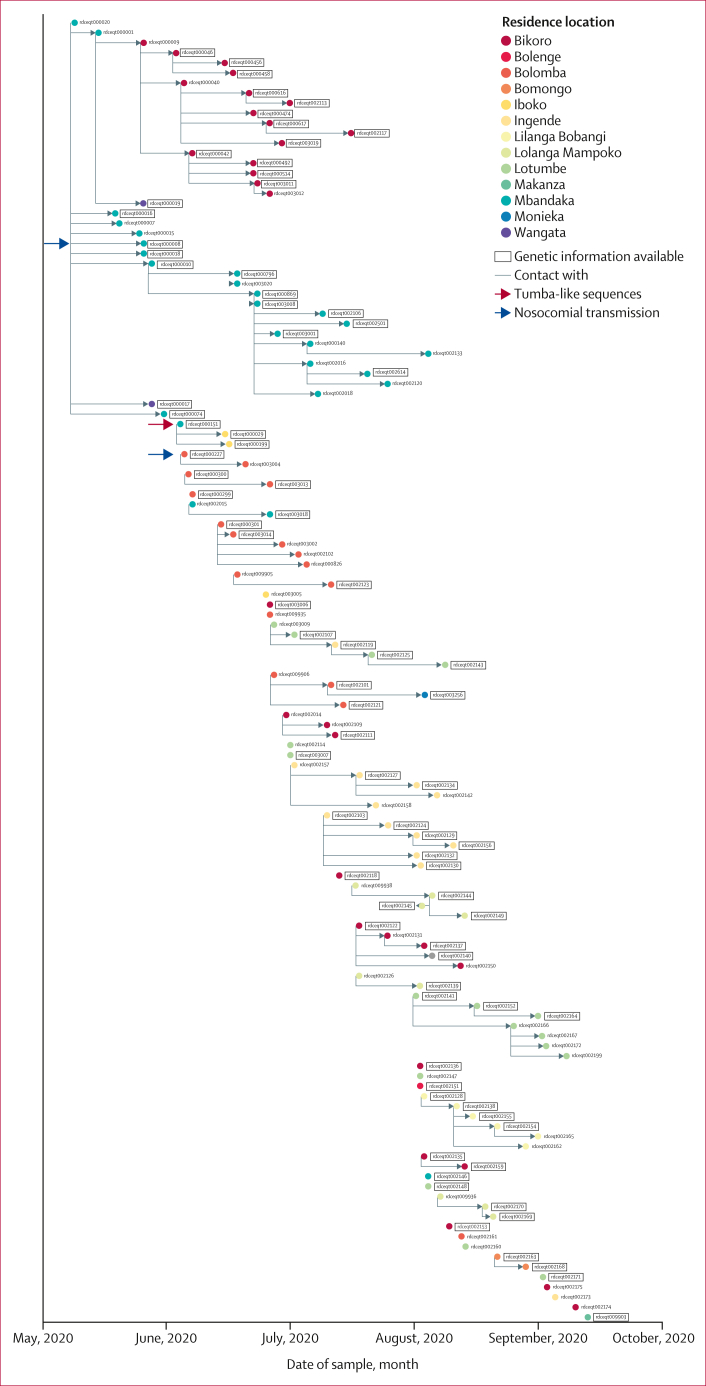
Social networks using interviews and contact tracing from the Équateur Province outbreak Each circle represents a single individual and is located at the date of symptom onset. Individuals from which genetic data are available are indicated with a box around the individuals’ identification number. Grey lines connecting nodes indicate social connections gathered from contact tracing interviews. The red arrow highlights the contact tracing chain associated with 2018 Tumba-like sequences. Grey arrows highlight examples of nosocomial transmission.

Between May 19 and Sept 16, 2020, 130 cases of EVD (119 laboratory-confirmed and 11 probable), were reported in 13 health zones (Bikoro, Bolenge, Bolomba, Bomongo, Iboko, Ingende, Lilanga Bobangi, Lolanga Mampoko, Lotumbe, Mkanza, Mbandaka, Monieka, and Wangata) of the Équateur Province in the Democratic Republic of the Congo (figure 1B). There was an overall case fatality rate of 42% (55 of 130 people died). The first EVD case symptom onset date was on May 19, 2020, and EVD cases peaked at the end of June and also the end of July (figure 2A). After August, 2020, the outbreak declined and the reported symptom onset date for the last case was on Sept 12, 2020. Men older than 45 years were the group with the most infections, whereas children aged 5–14 years had the least infections. This age distribution was different to the known age distribution for the population of the Democratic Republic of the Congo (figure 2B). The age distribution of people with EVD changed over time (figure 2C) and was different depending on location (appendix p 13). In the beginning of the outbreak (May to June, 2020), EVD cases occurred in individuals aged older than 15 years, whereas, from July to September, 2020, EVD was also recorded in children younger than 14 years (figure 2C). Symptom onset dates were available for 130 (100%) cases and community isolation dates were available for 98 (75%) cases, leading to the identification that the modal time from symptom onset to isolation was 6 days (appendix p 13). As initially seen with the probable first index case, 47 (36%) of 130 people with EVD visited more than one health-care facility after the onset of symptoms and three individuals visited up to four health-care facilities (figure 2D). The proportion of confirmed or probable cases who sought care at multiple health-care facilities decreased over time, with an average of 2.0 health-care facilities visited in May, which decreased to 1·5 in June, 1·6 in July, 1·6 in August, and 1·9 in September (figure 2D). The entire outbreak, including the known contact tracing chains and spread through different districts, can be observed in figure 3. The earliest identified probable case in this outbreak was a woman aged 37 years (who was a housewife) residing in the Mbandaka health zone who frequently consumed wild bat meat but had no contact with sick individuals, did not attend any funerals, and did not visit health centres in the period before her symptoms onset, suggesting that the outbreak probably started as a result of zoonotic spillover. The earliest identified probable case was linked to nine subsequent EVD infections, resulting in the spread of EVD in the Mbandaka and Wangata health zones (figure 3).

There were three health-care workers and one traditional healer among those who had EVD, two of whom died. Other commonly affected occupational groups included farmers, fishers, and hunters (37 [28%] of 130), housewives (25 [19%]), and business people (seven [5%]). The most common exposure risk reported by people with confirmed and probable EVD was having contact with another person with a confirmed or probable EVD case (94 [72%] of 130; table). 23 (18%) of 130 cases did not have a known epidemiological link, nine (7%) of 130 had a reported person-to-person link to an unidentified probable case, and four (3%) of 130 had a reported potential link with animals (table). The most likely settings of person-to-person transmission were in the community (20 [15%] of 130 people; ie, a non-family member, friend, or neighbour [or other interactions] outside of a health-care facility), in the individual’s household (19 [15%] people), or in the individual’s household or at a funeral (12 [9%]; table). 27 (21%) of 130 individuals reported multiple exposure locations and 44 (34%) of 130 did not report an exposure setting (unknown exposure).

**Table:** Summary of epidemiological links and transmission types from patient-reported data and case report forms

	Number of individuals (N=130)
Epidemiological link	
Person-to-person with a known confirmed or probable case	94 (72%)
Person-to-person with an unidentified probable case	9 (7%)
Animal	4 (3%)
Unknown link	23 (18%)
Type of person-to-person transmission	
Funeral	11 (8%)
Household	19 (15%)
Household or funeral	12 (9%)
Community	20 (15%)
Community or funeral	12 (9%)
Community or household	3 (2%)
Nosocomial	9 (7%)
Unknown exposure type	44 (34%)

Data are n (%).

**Figure 4:**
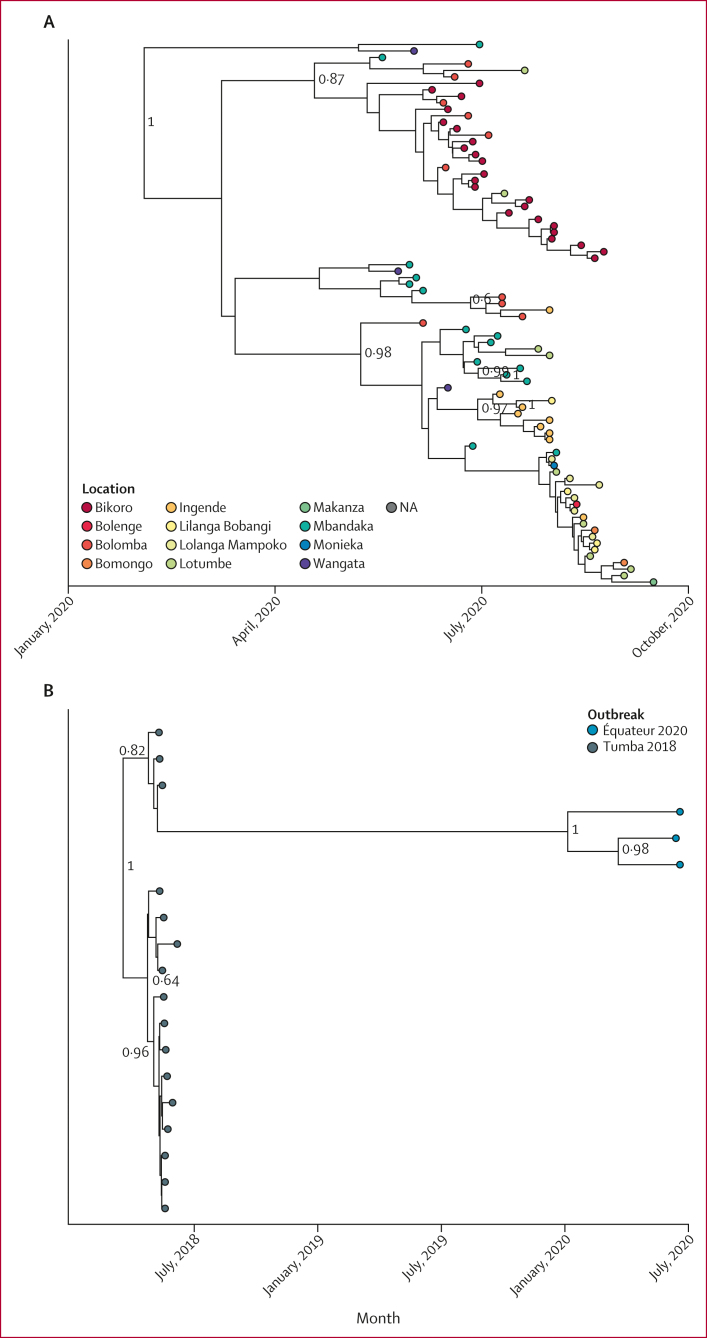

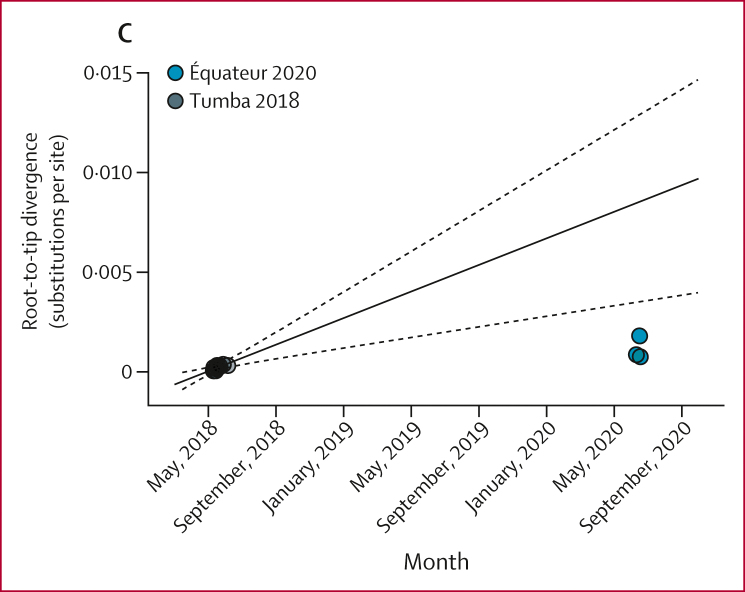
Ebola virus variants circulating during the 2020 Équateur Province outbreak (A) Inferred phylogenetic history using all available Mbandaka variant sequences. Posterior probabilities greater than 0·7 are labelled at internal nodes. (B) Inferred phylogenetic history of Tumba variant sequences. Posterior probabilities greater than 0·7 are labelled at internal nodes. (C) The genetic divergence from root versus collection date for the 2018 Équateur (Tumba variant) outbreak, including Tumba-like sequences from the 2020 Équateur Province EVD outbreak. The dashed line indicates the substitution rate for the 2018 Équateur outbreak and the dotted lines are 95% prediction intervals using only the 2018 Tumba sequences. EVD=Ebola virus disease. NA=not available.

We attempted next-generation sequencing using 188 specimens from 122 individuals with an epidemiological identifier and 31 individuals without an epidemiological identifier (specimens were not available from all 130 individuals with confirmed and probable EVD cases and, in some instances, multiple specimens were collected from the same patient). Overall, a total of 87 genomes were generated with sequencing coverage above 70% (appendix p 14). By sequencing these genomes, we found two circulating EBOV variants associated with the outbreak (figure 4A, B; appendix p 15). Of 87 total sequences, 84 (97%) were the Mbandaka variant and three complete genomes and one partial genome were the Tumba variant. The Mbandaka variant sequence data exhibited characteristics of one or two spillover events into the human population and the root age was estimated as Jan 13, 2020 (95% credible interval Dec 12, 2018, to June 2, 2020; figure 4A). The substitution rate for the Mbandaka variant was 0·474 × 10^–3^ (95% highest posterior density [HPD] 0·263–0·699 × 10^–3^) substitutions per site per year using Bayesian analysis and 0·818 × 10^–3^ (95% CI 0.391–1.245 × 10^–3^) substitutions per site per year using a regression analysis of root-to-tip distance over time (RTT; appendix p 16). In general, the two Mbandaka clades appeared to cluster geographically (figure 4A). Three sequences from this outbreak were similar to the 2018 Tumba variant from the Iboko and Bikoro health zones (figure 4A).^2^ These new sequences exhibited a delayed mutation rate of 0·443 × 10^–3^ (95% HPD 0·217–0·705) substitutions per site per year using Bayesian analysis compared with the acute substitution rate from the 2018 Tumba outbreak of 4·090 × 10^–3^ (1·24–7·07) substitutions per site per year using Bayesian and 3·836 × 10^–3^ (95% CI 1.584–6.087 × 10^–3^) substitutions per site per year using RTT methods (figure 4B, appendix p 16), which was consistent with transmission from a persistently infected survivor of the 2018 outbreak. The first identified Tumba-like EVD case was in a man aged 19 years who had no reported contact with any wild animals or bush meat, no contact with a sick individual, and no visits to a funeral or health facility and investigators were unable to confirm that he was persistently infected as a 2018 EVD case. After the onset of EVD symptoms, this individual visited two health-care facilities and an indistinguishable EBOV Tumba-like variant was detected in three subsequent cases with epidemiological links to the 19-year-old man (two complete genomes and one partial genome from three individuals).

**Figure 5:**
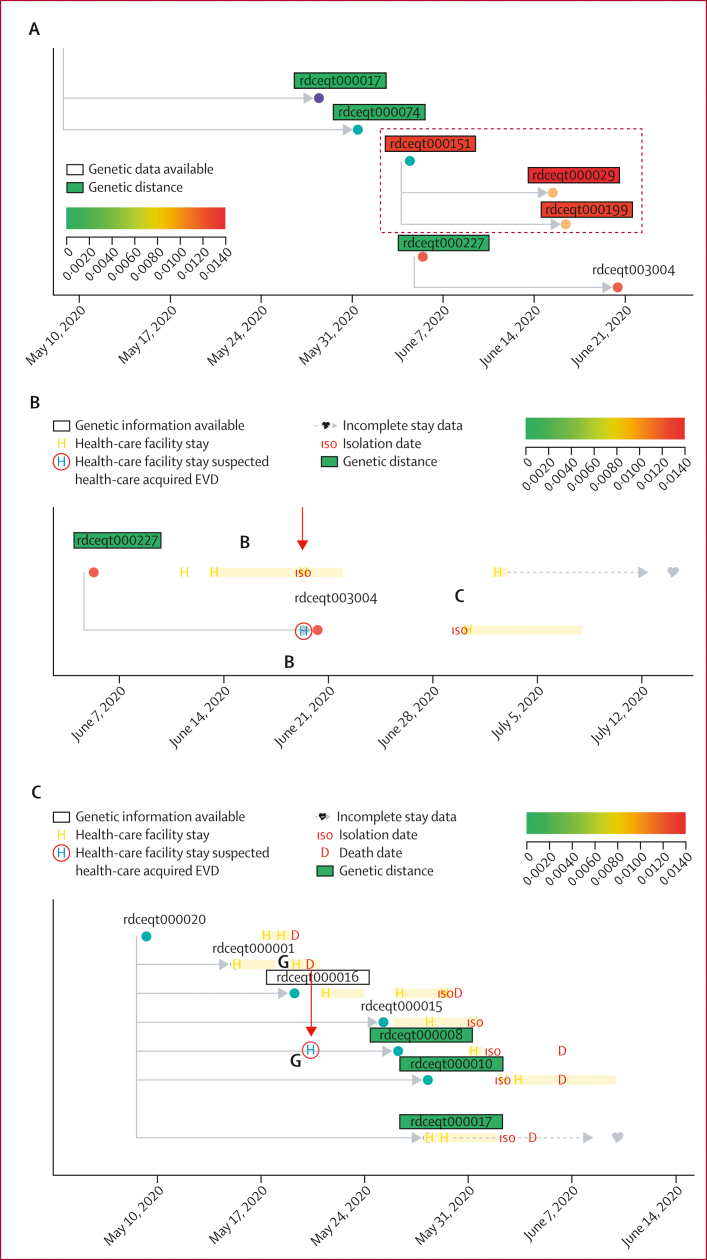
Epidemiological and phylogenetic networks showing details from the 2020 Équateur Province outbreak (A) Genetic data and contact tracing chains for co-circulating Mbandaka and Tumba EBOV variants. Filled circles represent single individuals and are located at symptom onset dates. Colours indicate residence location. The red box indicates a contact tracing chain containing Tumba-like EBOV sequences. Identification numbers with boxes around them show that genetic data were available, with shading in boxes indicating the genetic distance from the earliest sequence in the outbreak (with green showing little genetic distance and red showing greater genetic distance). (B) An example of nosocomial transmission from overlapping health-care facility stays (red arrow). Individual rdceqt003004 visited health-care facility B, concurrently with individual rdceqt000227 (who had EVD), before becoming positive for EVD. Filled circles represent single individuals and are located at symptom onset dates. Circle colours indicate residence location. Yellow bars represent length of stay at health-care facilities after EVD symptom onset. (C) Contact tracing data re-evaluated by combining genetic and epidemiological data. Metadata showing the identification of overlapping hospital stays at facility G between the individual rdceqt000001 (EVD case) and individual rdceqt000008 (red arrow). Individual rdceqt000008 subsequently developed EVD. Filled circles represent individuals and are located at symptom onset dates. Circle colours indicate residence location. Boxes around patient numbers indicate that genetic data were available and box colour (green) indicates close genetic relatedness between viral sequences relative to the sequence from individual rdceqt000016. EBOV=Ebola virus. EVD=Ebola virus disease.

Using patient-reported exposure and contact data, we constructed an extensive view of the EBOV outbreak in Équateur Province, including genetic data (when available), hospital visits (number, location, and duration) before and after EVD onset, date of symptoms onset, date of community isolation (either date of Ebola treatment unit [ETU] admission or date of death), and recovery status (appendix p 17). This plot documents the connected and unconnected epidemiological connections and viral geographical spread (figure 3) and overlays genetic data onto these epidemiological chains (appendix p 17). In general, there were 19 unconnected contact chains (containing two or more individuals) before genetic analysis. We used genetic data to make connections for 18 unconnected chains into the larger disease transmission chain (appendix p 17). 20 of 130 individuals were not connected to any contact tracing chains. 11 (55%) of these individuals could be added to the larger disease transmission chain using genetic data (the remaining nine [45%] individuals did not have associated genetic data). The visualisation also allowed the identification of the three Tumba-like sequences that were genetically distinct (figure 5A). Three individuals (not counting the individual identified as the index case) reported epidemiological spread from animals; however, the genetic data from each individual or their close contacts supported their inclusion in the ongoing Mbandaka outbreak, rather than being from zoonotic spillover of new EBOV variants (appendix p 17). Additional undocumented nosocomial cases were identified in which individuals without EVD symptoms visited health-care facilities concurrently with individuals identified as EVD cases and subsequently developed EVD (figure 5B, C). These two individuals (rdceqt003004 and rdceqt000008) were not among the nine individuals who had suspected nosocomial transmission cases initially reported from the contact tracing data (table).

## Discussion

In this study, we provide an extensive view of the 2020 Équateur Province EBOV outbreak by combining both genetic and epidemiological data. However, this integrated view is still likely to be incomplete, as some cases and contacts could only be identified as probable, suggesting that additional EVD cases might not have been fully documented, which was similar to observations from the tenth EVD outbreak in Nord Kivu Province, Democratic Republic of the Congo.^21^ In our epidemiological case investigations, we reported evidence that the 2020 Équateur Province EBOV outbreak started in the Mbandaka health zone and originated from an individual who frequently consumed bat meat and, using genetic data, we demonstrated that the EBOV sequence from the index case represented a new Ebola virus variant. Using additional genetic evidence, we demonstrated that a second EBOV variant re-emerged from an EVD survivor of the previous 2018 Équateur outbreak during the same timeframe. These data refuted the initial hypothesis that the Équateur Province outbreak was related to the ongoing EVD outbreak in the western Democratic Republic of the Congo in Nord Kivu Province.^18^ By combining the social and genetic datasets, a better understanding of the mechanisms of viral spread, exposure risk factors, and areas needing targeted improvement for future outbreak responses will be gained.

During the Équateur Province outbreak, there were several identified mechanisms of viral spread, including zoonotic, nosocomial, close-contact, and spread from a persistently infected EVD survivor. One challenge during this outbreak (and during more recent EVD outbreaks in Guinea^22^ and the Democratic Republic of the Congo in Nord Kivu Province^17^^,^^23^ and Équateur Province 2022^24^) had been identifying whether viral spread occurred due to re-emergence from a persistently infected EVD survivor or due to new zoonotic spillover. Distinguishing these nuances is especially challenging in an agrarian society, where individuals have frequent interactions with livestock or are consuming bushmeat. An increasing amount of evidence demonstrates that transmission from persistently infected survivors is indicated by a virus having close genetic relatedness to a previous outbreak as well as by exhibiting a delayed mutation rate.^10^^,^^11^^,^^13^^,^^16^ In contrast, viral genomes from a new zoonotic spillover generally appear to segregate into a distinct viral clade, such as in Équateur Province 2022^24^ or for a Marburg case in Ghana in 2022.^25^ Next-generation sequencing implemented in real time during an outbreak (initially through unbiased sequencing, followed by a targeted amplicon-based minION approach) and combined with epidemiological data using the ChainChecker tool is crucial to rapidly link and correct chains of transmission. These data indicate that rapid and retrospective genetic sequencing combined with epidemiological data is necessary during outbreak investigations because it directs responders to the mechanisms of viral spread and subsequent targeted interventions.

Studies from the 2014 west African EVD outbreak in Guinea, Liberia, and Sierra Leone have emphasised the need for survivor studies to educate survivors as to their potential ongoing EVD transmission risks,^26^^,^^27^ and those sentiments are also reinforced in this Article. During the 2020 Équateur outbreak, it took on average 6 days to isolate EVD cases from the community (either into an ETU or following death) and individuals with EVD visited multiple health-care facilities after the onset of symptoms (in some cases, up to four facilities). Therefore, rapid diagnostic testing,^28^ mobile laboratory testing, and post-mortem surveillance are needed to create a framework to rapidly identify and isolate future EVD cases. In Uganda, Shoemaker and colleagues^29^ demonstrated that an enhanced viral haemorrhagic fever surveillance programme—consisting of targeted viral haemorrhagic fever virus training at sentinel surveillance hospitals—decreased the duration and severity of viral haemorrhagic fever virus outbreaks.^28^ A similar viral haemorrhagic fever virus sentinel surveillance system located at sites of previous EVD outbreaks in the Democratic Republic of the Congo could also be used to rapidly identify and isolate future EVD cases. In addition, conducting multidisciplinary investigation in a One Health approach during future outbreaks might generate more consistent data for prevention and response.

In the last 3 weeks of the Équateur Province outbreak, new EVD cases were confined to six health areas, decreasing to three and then two health areas in the final 2 weeks. Eight of the 13 cases from the final 3 weeks of the outbreak were in children younger than 14 years, which possibly suggests that individuals older than 15 years had pre-existing antibodies due to previous EBOV outbreaks or vaccine-derived protection. During the 2020 Équateur Province outbreak, the rVSVΔG-ZEBOV-GP vaccine was administered in a ring vaccination strategy for individuals older than 6 months who were primary contacts of individuals with EVD and contacts of contacts. However, it is not known whether the individuals identified as the final EVD cases, or their contacts, were vaccinated. Retrospective studies from the Nord Kivu Province outbreak identified that vaccination with the rVSVΔG-ZEBOV-GP vaccine was associated with fewer EVD symptoms, reduced ETU stay, and decreased deaths^30^ and detected anti-glycoprotein antibodies in individuals at 21 days and 6 months after they had been vaccinated.^31^ It is currently unknown whether protective immunity from natural infection or vaccination wanes over time and whether this loss of protection correlates with EBOV re-emergence from persistently infected EVD survivors. Future serological surveys that can distinguish between natural-derived and vaccine-derived antibody responses will help address these questions.

Previous work during EVD outbreaks has focused on building genomic surveillance capacity in countries and developing a sustainable system.^2^ The paradigm of SARS-CoV2 genomic surveillance has also caused a significant shift in public health surveillance capacity in Africa.^32^ By improving the previous capacity-building efforts in the Democratic Republic of the Congo and consolidating the epidemiological and genetic datasets, we have presented a wide view of the Équateur Province 2020 EVD outbreak, allowing the identification of unforeseen trends and the fact-checking of pre-existing assumptions.

## Data sharing

An anonymised, de-identified version of the dataset can be made available on reasonable request to the corresponding author.

## Declaration of interests

We declare no competing interests.
