# Supplementary material for: 2020 Ebola virus disease outbreak in Équateur Province, Democratic Republic of the Congo: a retrospective genomic characterisation
Source: Lancet Microbe. 2024 Feb;5(2):e109–18. doi: 10.1016/S2666-5247(23)00259-8 (PMC10849974; doi:10.1016/S2666-5247(23)00259-8)
Supplement: Supplementary appendix [file mmc1.pdf]

THE LANCET
Microbe

Supplementary appendix

This appendix formed part of the original submission and has been peer reviewed.
We post it as supplied by the authors.

Supplement to: Kinganda-Lusamaki E, Whitmer S, Lokilo-Lofiko E, et al. 2020 Ebola virus disease outbreak in Équateur Province, Democratic Republic of the Congo: a retrospective genomic characterisation. *Lancet Microbe* 2024. https://doi.org/10.1016/S2666-5247(23)00259-8

Organisation mondiale de la Santé

RECOMMANDATION PROVISOIRE

Définitions de cas recommandées pour la surveillance des maladies à virus Ebola ou Marburg

9 Août 2014

1. Les définitions de cas standard

**Surveillance de routine**[1]	• **Cas présumé**<br>Toute personne souffrant d'une forte fièvre qui ne répond à aucun traitement des causes habituelles de fièvre dans la région et qui présente au moins l'un des signes suivants :<br>- diarrhée sanglante<br>- hémorragie gingivale<br>- hémorragies cutanées (purpura)<br>- injection des conjonctives<br>- présence de sang dans les urines<br><br>• **Cas confirmé**<br>Cas présumé confirmé par le laboratoire (sérologie positive des IgM, RT-PCR positive ou isolement du virus).
**Surveillance à base communautaire**	• **Case alerte**<br>a. Toute personne présentant une fièvre élevée à début brutal qui ne répond à aucun traitement des causes habituelles de fièvre dans la region ; **OU**<br>b. Toute personne ayant présenté une hémorragie ou une diarrhée sanglante ou une hématurie ; **OU**<br>c. Toute personne morte subitement.<br><br>• **Si un cas alerte (vivant ou décédé) est identifié,** le notifier à une équipe de surveillance ou au centre de santé le plus proche.<br><br>• Cette définition de "cas alerte" de maladie à virus Ebola ou Marburg a été élaborée pour être utilisée par la communauté et les relais communautaires. Elle peut être utilisée pour la surveillance à base communautaire dans la période pré-épidémique et pendant l'épidémie.

Remarque : Lors d'une flambée d'Ebola ou Marburg, il faut utiliser les définitions de cas présentées dans les section 2.

[1] Ces définitions de cas sont extraites du Guide technique pour la Surveillance Intégrée de la Maladie et la Riposte (SIMR) dans la Région Africaine, disponible à l'adresse suivante http://www.afro.who.int/groupes-organiques-et-programmes/ddc/surveillance-integree-de-la-maladie/

© Organisation mondiale de la Santé 2015.

Les appellations employées dans la présente publication et la présentation des données qui y figurent n'impliquent de la part de l'Organisation mondiale de la Santé aucune prise de position quant au statut juridique des pays, territoires, villes ou zones, ou de leurs autorités, ni quant au tracé de leurs frontières ou limites. Les traits discontinus formés d'une succession de points ou de tirets sur les cartes représentent des frontières approximatives dont le tracé peut ne pas avoir fait l'objet d'un accord définitif.

L'Organisation mondiale de la Santé a pris toutes les précautions raisonnables pour vérifier les informations contenues dans la présente publication. Toutefois, le matériel publié est diffusé sans aucune garantie, expresse ou implicite. La responsabilité de l'interprétation et de l'utilisation dudit matériel incombe au lecteur. En aucun cas, l'Organisation mondiale de la Santé ne saurait être tenue responsable des préjudices subis du fait de son utilisation.

WHO/EVD/CaseDef/14.1

2. Pendant la flambée de maladie à virus Ebola ou Marburg : définitions de cas

Définition de cas à utiliser par les équipes mobiles ou les postes et centres de santé

Important: *Lors d'une épidémie, les définitions de cas sont susceptibles d'être modifiées pour être adaptées à une nouvelle présentation clinique ou à des modes de transmissions différents liés à l'événement local.*

- **Cas suspect**
  a. Toute personne, vivante ou décédée, présentant ou ayant présenté une fièvre élevée à début brutal, et ayant été en contact avec :
    - un cas suspect, probable ou confirmé d'Ebola ou de Marburg ;
    - un animal mort ou malade (pour Ebola)
    - une mine (pour Marburg); **OU**

  b. Toute personne présentant une fièvre élevée à début brutal et au moins trois des symptômes suivants :
    - maux de tête
    - anorexie / perte d'appétit
    - douleurs abdominales
    - vomissements
    - diarrhée
    - fatigue intense
    - difficultés à avaler
    - difficultés à respirer
    - hoquet
    - douleurs musculaires ou articulaires ; **OU**

  c. Toute personne présentant des saignements inexpliqués ; **OU**
  d. Toute personne morte subitement et dont le décès est inexpliqué

- **Conduite à tenir lorsqu'un cas suspect a été identifié**
  - Notifier le cas à l'équipe de surveillance
  - Après consentement exprès, collecter un échantillon biologique
  - Remplir une fiche de notification de cas
  - Dresser la liste des contacts du cas suspect
  - Si le sujet est vivant, il faut expliquer au patient et à sa famille la nécessité d'aller à l'hôpital pour recevoir les soins médicaux appropriés. Après avoir obtenu le consentement du patient ou de sa famille il faut organiser son transport.
  - Si le sujet est décédé, il faut expliquer à la famille la nécessité de conduire un enterrement sécurisé. Après consentement, il faut coordonner avec l'équipe enterrement l'organisation des funérailles.

Définition de cas à utiliser uniquement au niveau des hôpitaux et des équipes de surveillance

Important: *lors d'une épidémie, les définitions de personnes contact sont susceptibles d'être modifiées pour être adaptées à de nouveaux facteurs de risques d'infection liés à l'événement local.*

- **Cas probable**
  a. Tout cas suspect évalué par un clinicien ; **OU**
  b. Tout cas suspect décédé (et pour lequel il n'a pas été possible d'obtenir des échantillons biologiques pour confirmation au laboratoire) ayant un lien épidémiologique avec un cas confirmé

  Note: si les échantillons de laboratoire sont prélevés en temps opportun pendant la maladie, les catégories précédentes sont reclassées comme cas « confirmés au laboratoire » et « non cas ».

- **Cas confirme au laboratoire**
  Tout cas suspect ou probable avec un résultat de laboratoire positif. Les cas confirmés au laboratoire doivent être positifs soit pour l'antigène du virus, soit pour l'ARN viral détecté par transcription inverse suivie de la réaction en chaîne par polymérase (RT- PCR), soit pour les anticorps IgM dirigés contre Marburg ou Ebola.

- **Non-cas**
  Tout cas suspect ou probable avec un résultat de laboratoire négatif. Les « non-cas » étaient dépourvus d'anticorps spécifiques, d'ARN et d'antigènes spécifiques décelables.

Définition standard des personnes contacts de cas d'Ebola ou de Marburg

***Important:** lors d'une épidémie, les définitions de personnes contact sont susceptibles d'être modifiées pour être adaptées à de nouveaux facteurs de risques d'infection liés à l'événement local.*

- **Personne contact d'un cas d'Ebola ou de Marburg**
  Toute personne ayant été exposée à un cas suspect, probable ou confirmé d'Ebola ou de Marburg selon au moins une des modalités suivantes, a condition que cette exposition ait eu lieu moins de 21 jours avant l'identification comme contact par les équipes de surveillance :
  - a dormi dans le même foyer que le cas
  - a eu un contact physique direct avec le cas (vivant ou décédé) pendant sa maladie
  - a eu un contact physique direct avec le cas (décédé) pendant les funérailles
  - a eu un contact direct avec le sang ou les fluides corporels du cas pendant sa maladie
  - a eu un contact direct avec les vêtements ou le linge du patient
  - touched his/her clothes or linens
  - a été allaité au sein d'un cas (pour un bébé)

- **Personne contact d'un animal mort ou malade**
  Toute personne ayant été exposée à un animal décédé ou malade selon au moins une des modalités suivantes, a condition que cette exposition ait eu lieu moins de 21 jours avant l'identification comme contact par les équipes de surveillance :
  - a eu un contact physique direct avec l'animal
  - a eu un contact direct avec le sang ou les fluides corporels de l'animal
  - a dépecé l'animal
  - a mangé de la viande de brousse crue

- **Personne contact d'un laboratoire**
  Toute personne ayant été directement exposée à du matériel biologique dans un laboratoire selon au moins une des modalités suivantes, a condition que cette exposition ait eu lieu moins de 21 jours avant l'identification comme contact par les équipes de surveillance :
  - a eu un contact direct avec des prélèvements de patients suspect, probable ou confirmé d'Ebola ou de Marburg
  - a eu un contact direct avec des prélèvements d'animaux suspects d'Ebola ou de Marburg

- **Les autres facteurs de risques d'infection incluent :** contact avec un établissement hospitalier où des cas d'Ebola ou de Marburg ont été pris en charge ; injection ou vaccination dans les 21 jours précédant le début des symptômes.

- **La personne contact doit être suivi pendant 21 jours après l'exposition.** Si le contact est asymptomatique pendant 21 jours après son exposition, il sort du suivi.

FICHE DE NOTIFICATION DE FIEVRE HEMORRAGIQUE VIRALE

Numéro d'identification du patient: ____________

Autre numéro d'identification : ____________

Date de Notification: ____/____/_____ (J, M, A)

Section 1. Information sur le Patient

Nom de Famille: ________________________ Autres Noms:_______________________________ Age: _______ ☐ Années ☐ Mois

Sexe: ☐ Masculin ☐ Féminin Numéro de Téléphone (Patient/Famille):_________________ Propriétaire du Téléphone: _____________

Etat du patient au moment de la collecte d'information: ☐ Vivant ☐ Décédé *Si décédé*, Date du Décès: ___/___/____ (J, M, A)

Lieu de Résidence Permanente:

Nom du Chef de Famille: __________________________ Village/Quartier : _____________________Zone de Santé : _______________

Pays de Résidence: _____________________ Province:________________________ Aire de Santé: __________________________

Occupation:

☐ Planteur/Eleveur ☐ Boucher ☐ Chasseur/Vendeur de viande de brousse ☐ Mineur ☐ Chef religieux

☐ Ménagère ☐ Etudiant ☐ Enfant ☐ Médecin traditionnel

☐ Commerçant; type de commerce: ________________________ ☐ Transporteur; type de transport: ___________________________

☐ Personnel de santé; position: ________________________ nom du centre médical:______________________________

☐ Autre; précisez le métier: ____

Endroit où le Patient est Tombé Malade:

Village/Quartier: __________________________ Zone de santé : _________________________ Aire de Santé : __________________

Coordonnées GPS de la maison: Latitude:________________________ Longitude: _______________________

Si ce n'est pas la résidence permanente, Dates de résidence dans cet endroit: ___/__/____ - ___/__/____ (J, M, A)

Section 2. Signes Cliniques et Symptômes

Date de début des signes et symptômes: ____/____/_____ (J, M, A)

Cochez tous les signes et symptômes observés ou ressentis entre la date de début de la maladie et la date de notification:

Fièvre	☐ Oui	☐ Non	☐ Inc
Si oui, Temp: ____º C Mesure: ☐ Creux Axillaire ☐ Bouche ☐ Rectale			
Nausées / Vomissements	☐ Oui	☐ Non	☐ Inc
Diarrhées	☐ Oui	☐ Non	☐ Inc
Fatigue générale intense	☐ Oui	☐ Non	☐ Inc
Perte d'appétit / anorexie	☐ Oui	☐ Non	☐ Inc
Douleurs abdominales	☐ Oui	☐ Non	☐ Inc
Douleurs thoraciques	☐ Oui	☐ Non	☐ Inc
Douleurs musculaires	☐ Oui	☐ Non	☐ Inc
Douleurs articulaires	☐ Oui	☐ Non	☐ Inc
Céphalées	☐ Oui	☐ Non	☐ Inc
Toux	☐ Oui	☐ Non	☐ Inc
Difficultés à respirer	☐ Oui	☐ Non	☐ Inc
Difficultés à avaler	☐ Oui	☐ Non	☐ Inc
Mal à la gorge	☐ Oui	☐ Non	☐ Inc
Ictère (conjonctives/gencives/peau)	☐ Oui	☐ Non	☐ Inc
Conjonctivite (œil rouge)	☐ Oui	☐ Non	☐ Inc
Eruptions cutanées	☐ Oui	☐ Non	☐ Inc
Hoquet	☐ Oui	☐ Non	☐ Inc
Douleurs retro-orbitaires/photophobie	☐ Oui	☐ Non	☐ Inc
Coma / perte de conscience	☐ Oui	☐ Non	☐ Inc
Confusion ou désorientation	☐ Oui	☐ Non	☐ Inc

**Saignements**	☐ Oui	☐ Non	☐ Inc
Si Oui:			
Saignements des gencives	☐ Oui	☐ Non	☐ Inc
Saignements aux sites d'injections	☐ Oui	☐ Non	☐ Inc
Saignements du nez (épistaxis)	☐ Oui	☐ Non	☐ Inc
Selles rouges ou noires (mélénas)	☐ Oui	☐ Non	☐ Inc
Vomissements sanglants (hématémèses)	☐ Oui	☐ Non	☐ Inc
Vomissement noirâtre (vomito negro)	☐ Oui	☐ Non	☐ Inc
Toux sanglante (hémoptysie)	☐ Oui	☐ Non	☐ Inc
Saignements vaginaux, en dehors des règles	☐ Oui	☐ Non	☐ Inc
Hématomes / Pétéchies / purpura	☐ Oui	☐ Non	☐ Inc
Sang dans les urines (hématurie)	☐ Oui	☐ Non	☐ Inc
Autres signes hémorragiques	☐ Oui	☐ Non	☐ Inc

Si oui, précisez: ___________________________

Autres signes cliniques non-hémorragiques: ☐ Oui ☐ Non ☐ Inc

Si oui, précisez: ___________________________

Section 3. Informations sur l'hospitalisation

Au moment de cette notification, le malade est-il déjà hospitalisé ou en cours d'admission à l'hôpital? ☐ Oui ☐ Non

Si oui, Date d'hospitalisation: ____/____/_____ (J, M, A) Nom de l'hôpital: ____

Village/Ville: _______________________ Zone de Santé: ______________________ Aire de Santé: ____________________

Le malade est-il en isolement/en cours d'isolement? ☐ Oui ☐ Non *Si oui, D*ate d'isolement: ____/____/_____ (J, M, A)

Le malade était-il hospitalisé ailleurs ou a visité un centre de soins pour la maladie actuelle? ☐ Oui ☐ Non ☐ Inc

Si oui, veuillez compléter une ligne ci-dessous pour chacune des hospitalisations précédentes:

Dates d'hospitalisation	Nom du centre en médical	Village	Zone de santé	Le patient était-il isolement?
___/___/____ - ___/___/____ (J, M, A)				☐ Oui<br>☐ Non
___/___/____ - ___/___/____ (J, M, A)				☐ Oui<br>☐ Non

Numéro d'identification du patient:

Section 4. Epidémiologie / Facteurs d'expositions

PENDANT LE MOIS PRÉCÉDENT LE DÉBUT DES SYMPTÔMES:

1. Il y a-t-il eu contacts avec un malade Ebola, connu/suspect, ou simplement avec une personne malade? ☐ Oui ☐ Non ☐ Inc

Si oui, veuillez compléter une ligne ci-dessous pour chacun des malades pouvant être une source de contamination:

Nom du malade potentiel	Lien de parenté	Date(s) du contact (J, M, A)	Village	Zone de santé	Est-ce-que la personne était vivante ou décédée ?	Types de contact**
		___/___/___ - ___/___/___			☐ Vivante<br>Décédée, Date: ___/___/____ (J, M, A)	
		___/___/___ - ___/___/___			☐ Vivante<br>Décédée, Date: ___/___/____ (J, M, A)	
		___/___/___ - ___/___/___			☐ Vivante<br>Décédée, Date: ___/___/____ (J, M, A)	

****Type de contact: (indiquez toutes les possibilités)**

1 – A touché des secrétions/excrétions du malade (sang, vomissures, salive, urine, selles)
2 – A touché directement le corps du malade (vivant ou décédé)
3 – A touché ou partagé linges, habits, plats/assiettes, instruments avec le malade
4 – A dormi ou mangé avec, ou séjourné dans la même maison ou pièce que le malade

2. Est-ce-que le patient a participé à des funérailles avant la maladie actuelle? ☐ Oui ☐ Non ☐ Inc

Si oui, veuillez compléter une ligne ci-dessous pour chacune des participations à un enterrement:

Nom de la personne décédée	Lien de parenté	Dates de participation aux funérailles (J, M, A)	Village	Zone de Santé	Avez-vous porté ou touché le corps?
		___/___/____ - ___/___/____			☐ Oui ☐ Non
		___/___/____ - ___/___/____			☐ Oui ☐ Non

3. Le patient a-t-il voyagé en dehors de chez lui ou de son village/ville avant la maladie actuelle? ☐ Oui ☐ Non ☐ Inc

Si oui, Village: ___________________________ Zone de santé : ___________________ Date(s): ___/___/____ - ___/___/____ (J, M, A)

4. Le patient a-t-il été hospitalisé, a-t-il consulté dans un hôpital ou visité quelqu'un hospitalisé avant la maladie actuelle? ☐Oui ☐Non ☐Inc

Si oui, Nom du patient: ____________________ Date(s): ___/___/____ - ___/___/____ (J, M, A)

Nom du Centre Médical: ________________________ Village: ______________________ Zone de Santé: ________________

5. Le patient a-t-il consulté un médecin traditionnel avant la maladie actuelle? ☐ Oui ☐ Non ☐ Inc

Si oui, Nom du Médecin: ________________Village: _______________ Zone de santé: _____________ Date: ___/___/____ (J, M, A)

6. Le patient a-t-il eu un contact direct (chasse, touché, mangé) avec des animaux ou de la viande crue avant de tomber malade? ☐Oui ☐Non ☐Inc

Si oui, cochez les cases nécessaires:

Animal:	Status (check one only):
☐ Chauve-souris (ou excrétions de)	☐ En bonne santé ☐ Malade/Mort
☐ Singes	☐ En bonne santé ☐ Malade/Mort
☐ Rongeurs (ou excrétions de)	☐ En bonne santé ☐ Malade/Mort
☐ Cochons	☐ En bonne santé ☐ Malade/Mort
☐ Volaille ou oiseaux sauvages	☐ En bonne santé ☐ Malade/Mort
☐ Vaches, chèvres, ou moutons	☐ En bonne santé ☐ Malade/Mort
☐ Autres; *précisez :*_____________	☐ En bonne santé ☐ Malade/Mort

7. Est-ce-que le patient a eu une piqûre de tique dans les 2 dernières semaines? ☐ Oui ☐ Non ☐ Inc

Section 5. Prélèvements Biologiques pour le Laboratoire

Mode de prélèvements et d'envoi :

- Identifier le tube: **nom**, **date de prélèvement** et le **numéro d'identification du malade**
- Envoyer les échantillons avec **réfrigération,** and **emballés correctement**.
- Prélever le sang complet dans un tube EDTA (bouchon violet) tube – si non disponible, bouchon vert (héparine) ou rouge (sans anticoagulant) sont acceptables
- **Volume demandé = 4ml** (volume minimum = 2ml)

Est-ce qu'un prélèvement a déjà été soumis pour ce malade? ☐ Oui ☐ Non

Prélèvement 1: *Ne pas remplir*

Date du prélèvement: ____/____/______ (J, M, A)

Type de prélèvement:

☐ Sang complet
☐ Ponction cardiaque (*post-mortem)*
☐ Biopsie de peau
☐ Autre prélèvement, précisez: ________________

Prélèvement 2: *Ne pas remplir*

Date du prélèvement: ____/____/______ (J, M, A)

Type de prélèvement:

☐ Sang complet
☐ Ponction cardiaque (*post-mortem)*
☐ Biopsie de peau
☐ Autre prélèvement, précisez: ________________

Section 6. Fiche de notification complétée par:

Nom: ______________________________ Téléphone: _________________________ E-mail: ____________________________Role:

ZS : ________________________ Centre médical: ____

Informations fournies par ☐ Patient ☐ Représentant; *Si représentant*, Nom:______________________ Lien de parenté: _____________

Nom du Patient: [ ]

Numéro Identification du Patient: [ ]

***Si le patient est décédé ou est déjà convalescent ou guéri, veuillez remplir la section suivante.**
***Si le malade va être admis à l'hôpital, ne complétez pas la section suivante, (ce sera fait lors de la sortie)**

Section 7. Statut final du patient

Veuillez remplir cette section lorsque le patient est guéri et sort de l'hôpital ou lors de son décès.

Date à laquelle les informations sont rapportées: ____/___/_____ (J, M, A)

Statut final du patient: ☐ Vivant ☐ Décédé

Est-ce-que le patient a eu des signes hémorragiques inexpliqués pendant la durée de la maladie? ☐ Oui ☐ Non ☐ Inc

Si oui, veuillez préciser: ____________________

Si le malade est guéri et sort de l 'hôpital :

Nom de l'hôpital: ____________________ Zone de Santé: ____________________

Si le malade était en isolement, date de sortie de la zone d'isolement: ____/___/______ (J, M, A)

Date de sortie de l'hôpital: ____/____/______ (J, M, A)

Si le malade est décédé:

Date du décès: ____/____/______ (J, M, A)

Lieu du décès: ☐ Domicile ☐ Hôpital: ____________________ ☐ Ailleurs: ____________________

Village : ____________________ Zone de santé: ____________________ Aire de santé: ____________________

Date des funérailles: ____/____/______ (J, M, A) Funérailles organisées par: ☐ Famille/communauté ☐ Equipe d'enterrement

Lieu des funérailles/enterrement:

Village: ____________________ Zone de Santé: ____________________ Aire de Santé: ____________________

Veuillez cocher une réponse pour tous les signes et symptômes, indiquant s'ils ont été trouvés ou non pendant toute la durée de la maladie (en tenant compte également de la période d'hospitalisation):

Fièvre	☐ Oui	☐ Non	☐ Inc
Si oui, Temp: ____º C Mesure: ☐ Creux Axillaire ☐ Bouche ☐ Rectale			
Nausées / Vomissements	Oui	Non	Inc
Diarrhées	☐ Oui	☐ Non	☐ Inc
Fatigue générale intense	Oui	Non	Inc
Perte d'appétit / Anorexie	☐ Oui	☐ Non	☐ Inc
Douleurs abdominales	Oui	Non	Inc
Douleurs thoraciques	☐ Oui	☐ Non	☐ Inc
Douleurs musculaires	Oui	Non	Inc
Douleurs articulaires	☐ Oui	☐ Non	☐ Inc
Céphalées	Oui	Non	Inc
Toux	☐ Oui	☐ Non	☐ Inc
Difficultés à respirer	Oui	Non	Inc
Difficultés à avaler	☐ Oui	☐ Non	☐ Inc
Mal à la gorge	Oui	Non	Inc
Ictère (conjonctives/gencives/peau)	☐ Oui	☐ Non	☐ Inc
Conjonctivite (œil rouge)	Oui	Non	Inc
Eruptions cutanées	☐ Oui	☐ Non	☐ Inc
Hoquet	Oui	Non	Inc
Douleurs retro-orbitaires/photophobie	☐ Oui	☐ Non	☐ Inc
Coma / perte de conscience	Oui	Non	Inc
Confusion ou désorientation	Oui	☐ Non	☐ Inc

Autres signes ou symptômes cliniques non hémorragiques: ☐ Oui ☐ Non ☐ Inc

Si oui, Veuillez précisez: ____________________

SUPPLEMENTAL EXPERIMENTAL PROCEDURES:

Case investigation and contact tracing

The DRC Ministry of Health's Ebola virus disease case definition was used to classify cases as suspected, probable, or confirmed (Supplemental File 1). The case definition used for a suspect case was different than that used in previous outbreaks in one significant way: fever was not a required sign, because fever is not uniformly present in all confirmed cases of EVD at the time of detection.

All suspect, probable and confirmed cases were investigated to record demographic characteristics, determine possible exposures, document information about illness onset and signs and symptoms, and to identify potentially exposed contacts. Data were collected by trained field investigators and health professionals using a standardized case investigation form (Supplemental File 2), which was subsequently entered into an electronic database. For suspect cases who recovered or died before the index case, retrospective case classification was performed by reviewing medical records at health facilities in the affected locations. For surviving suspect cases or for those whose disease onset was after the declaration of the outbreak on June 1, 2020, information was collected prospectively at the time of case investigation. Suspected cases reported throughout the study period were systemically investigated, sampled, and reclassified on the basis of laboratory results to confirm or exclude them as non-cases. Contacts were identified during the case investigation process for each case, and contact tracing began with the first field investigation (June 1, 2020). Contact tracers are required to visit all contacts twice a day for 21 days after the last date of contact with a probable or confirmed case. During each visit, information was collected with respect to the health status of contacts and the development of EVD-like symptoms.

Bioinformatics

Consensus genomes were constructed using the bioinformatics method appropriate to the library construction method – either the Artic EBOV bioinformatics protocol (for amplicon-based MinIon sequencing), or a read mapping to a reference genome using in-house scripts (https://github.com/evk3/EBOV_Equateur_2020) (for TruSeq-based Illumina sequencing). For the artic EBOV bioinformatics protocol, config files were changed to trim EBOV Equateur primer pools from the reads. For TruSeq-based Illumina sequencing, Illumina adaptors were removed from the sequencing reads using Cutadapt v1.21 and low quality reads/bases were filtered using Prinseq-lite v0.20.4 (-min_qual_mean 25 -trim_qual_right 20 -min_len 50). EBOV genomes were assembled by aligning trimmed reads to an Equateur reference sequence with bwa mem and new consensus genomes were called using samtools mpileup (-A -aa -d 6000000 -B -Q 0) and ivar consensus (1.3.1) (-m 2 -n N). Genomes acquired from clinical specimens were deposited into Genbank: OR084846-932. Multiple specimens were re-sequenced using MinIon and enrichment-based methods. In most cases sequences were identical, but differed only in the amount of genome coverage. Only a single sequence was kept for each individual and sequences were selected based on those that had most complete coverage. In limited instances indels at homoploymers sites disrupted open reading frames and these sites were adjusted by hand after reviewing the mapping files.

Phylogenetic and Bayesian Analysis

Viral genomes were aligned using mafft (version 7.450) and maximum likelihood trees were constructed using raxml (version 7.3.0-PTHREAD) with 1000 bootstrap iterations. Evolutionary rates were estimated using both linear regression modeling and time-scaled phylogenies. Linear regression modeling was performed using TempEst with maximum likelihood trees rooted on the

earliest available Mbandaka 2020 or Tumba 2018 sequences and genetic distance versus collection date was visualized in R (4.1.3). Evolutionary rates estimates, R^2 and p-values were calculated in R using lm(distance~date) with 95% prediction intervals estimated from the same dataset. Bayesian analysis was performed using Beast (1.10.4) with separate Mbandaka 2020 (including closely related sequences from 2014 and 2018 DRC outbreaks) or Tumba 2018 sequence sets. The MCMC analysis was conducted for 400 million generations, which represents a compilation of 4-independent replicates of 100 million generations (sampled every 10,000th state). Convergence was obtained for all replicates and burn-in was removed (5-10% of total states) by examining the trace and effective sample size statistics (min ESS > 200 for all models) using tracer/v1.7.1. Rate estimates were modeled using unlinked coding and non-coding HKY nucleotide substitution models with 4-independent Γ distributions. Model comparisons were conducted using different clock and population models with the Mbandaka 2020 sequences: Bayesian skygrid demographic model (with variable population model estimated between September 2013- 2020 (7 years and 50 grid points) or constant population and fixed local clock or uncorrelated lognormal local clock set with an initial prior of $1.1*10^{-3}$ subs/site/year. Bayesian analysis of the *Zaire ebolavirus* Tumba variant sequences was conducted using the Bayesian skygrid demographic model (with variable population model estimated between April 6, 2018 and June 18, 2020, ie – "Time at last point:2.2" over 50 grid points) and uncorrelated lognormal local clock set with an initial prior of $1.1*10^{-3}$ subs/site/year. Strength of model fit was evaluated by performing path- and stepping stone-sampling with default values and best-of-fit was evaluated by calculating Bayes Factors. Statistically strong differences in Bayes Factor values for different models were not observed (Supplemental Table 2), and the UCLN Skygrid model results were used to maintain uniformity with previous EBOV rate estimates. Substitution rate estimates were calculated using all available

Mbandaka sequences, only Tumba 2018 sequences, or only the branch leading to the Tumba-like re-emerged EBOV variant using custom-modified samogitia.py scripts (https://github.com/evk3/EBOV_Equateur_2020).

Quantification and Statistical Analysis

Geographic analysis was performed using QGIS (3.16.13) and choropleth maps were made using R (4.1.2). Analysis of epidemiological metadata was done using Python (3.9.13) in a Jupyter notebook (6.4.12). The DRC population demographic data was accessed from the WHO Demographic Yearbook – 2020 (71st Issue). MCC time-scaled phylogenies were visualized using ggtree (version 3.2.0), root-to-tip versus specimen collection dates were visualized using R (4.1.3) in a Jupyter notebook (6.4.12). Epidemiological and genetic data were visualized in ChainChecker version 2.0 (https://github.com/jhuapl-globalhealth/chainchecker/). Animal silhouettes were from PhyloPic using the Creative Commons Attribution 3.0 Unported license, this is attributed the work to Kai R. Caspar and Melissa Ingala.

Supplemental Table 1: List of Equateur-specific EBOV amplicon-based NGS primers.

primer name	primer sequence	pool
EBOV-400_01_LEFT	AGAATTTTTAGGATCTTTTGTGTGCGA	1
EBOV-400_01_RIGHT	CCCCAAGCTTTAGGGTTGTTGA	1
EBOV-400_03_LEFT	ATACAGGCCTTTGAAGCAGGTG	1
EBOV-400_03_RIGHT	AGCTGTTGGATATTGTATCAGTCCT	1
EBOV-400_05_LEFT	GTCTCCATCCTCTTGCAAGGAC	1
EBOV-400_05_RIGHT	AGTTACCATAGCGTTTGTTTGCTG	1
EBOV-400_07_LEFT	CGGCGAATACCAGAGTTACTCG	1
EBOV-400_07_RIGHT	TTCCGTCCCTGTCCTGTTCTTC	1
EBOV-400_09_LEFT	AGTTTTCAGTACCAGTGATGGCA	1
EBOV-400_09_RIGHT	AACGCCTATTTGCATTAAGACTCAA	1
EBOV-400_11_LEFT	TGAATTCTCTAGCACTCGAAGCT	1
EBOV-400_11_RIGHT	TCAAGACTCGTAATGCGTTGTTCT	1
EBOV-400_13_LEFT	GACCGTCCCTCAAAGTGTTAGG	1
EBOV-400_13_RIGHT	CCCAACCTCGATCAATCTTGGG	1
EBOV-400_15_LEFT	AGGAGCTATATCTCTGACAGTATTATAATCA	1
EBOV-400_15_RIGHT	CTTTGGGGCCCGATATGACATT	1
EBOV-400_17_LEFT	GCCAGTCCAACTACCCCAGTAT	1
EBOV-400_17_RIGHT	GGGTCCAAACCGATGTACTTTG	1
EBOV-400_19_LEFT	ATCCGTCCATTAGAGGAGATACTTTT	1
EBOV-400_19_RIGHT	GATTACGCTCACTGTCGGCTTA	1
EBOV-400_21_LEFT	TCATCCACAATAGCACATTACAGGT	1
EBOV-400_21_RIGHT	AACTGTGGAAGCAAGTCGATCA	1
EBOV-400_23_LEFT	AAGATTCACACCACAGTTTCTGC	1
EBOV-400_23_RIGHT	GGTTGAGGACTCGTGGAGATTG	1
EBOV-400_25_LEFT	TCCCCAAAACCACAGCGAGA	1
EBOV-400_25_RIGHT	CGGTTGAGGATTGAAAAGGTGC	1
EBOV-400_27_LEFT	GGCGTTATAATTGCAGTTATCGCT	1
EBOV-400_27_RIGHT	TCTACCGGTTCGCAGATCTGAT	1
EBOV-400_29_LEFT	CCTCACAAGTACGCGTTCCTAC	1
EBOV-400_29_RIGHT	AACTTCCTCGTCATCACAGCAC	1
EBOV-400_31_LEFT	TGCTGTCGTTGTTTCAGGGTTA	1
EBOV-400_31_RIGHT	GATGGGATGGATCGTTGCTACC	1
EBOV-400_33_LEFT	TGCGGAGGTCTGATAAGAATAAACC	1
EBOV-400_33_RIGHT	TGTCTAGACACTCTCAGTTCAACC	1
EBOV-400_35_LEFT	AACTAATGACTTTGCCCCTGCA	1
EBOV-400_35_RIGHT	ACCAGAAAACCCATGTTAGTTCGA	1
EBOV-400_37_LEFT	CTTGACATCTCTGCTTTCATAATCAGA	1
EBOV-400_37_RIGHT	TCAACTGCTTCATCTAAAACACGG	1
EBOV-400_39_LEFT	ACGATGTAACTGTTACCAAGTTCTTG	1
EBOV-400_39_RIGHT	ACATAGTCCCCATAACCTAAGATGTC	1
EBOV-400_41_LEFT	AGGATCCAGTTTGTTCTGATTATCCC	1
EBOV-400_41_RIGHT	TGCTTTTAGCACCGTAGCATGT	1
EBOV-400_43_LEFT	ACCATCCTCCACTTTTCTCAACC	1
EBOV-400_43_RIGHT	TTGCTCACGTTCCGTAACTACC	1
EBOV-400_45_LEFT	TCCACCACATAACCTCACACTG	1
EBOV-400_45_RIGHT	AGGCAATTGGACCCCATTCAAA	1
EBOV-400_47_LEFT	CGGTAAACCTCTGGATTTCGGA	1
EBOV-400_47_RIGHT	AACAGGAGTTGATGATAATAGCCATTT	1
EBOV-400_49_LEFT	ACTTGCACAGTTGATTTAGCACA	1
EBOV-400_49_RIGHT	TCACTGTTCGAACTGCCTTGAG	1
EBOV-400_51_LEFT	TCTGCACGCGACAGCAATATTA	1
EBOV-400_51_RIGHT	TCCACTGCTAATTGGGTCTGTAG	1
EBOV-400_53_LEFT	GCGTTATGTCACGGTTAATGAGT	1
EBOV-400_53_RIGHT	GCCAAGTATTTTCGGTTGCTGTT	1
EBOV-400_55_LEFT	TGTGGTACAGCAAATCCAAAACTAA	1
EBOV-400_55_RIGHT	GGACTCAGTAGCTAGCGTGTTG	1
EBOV-400_57_LEFT	ACAGATCGAAATTGTACGAAGCTG	1
EBOV-400_57_RIGHT	ACCTGTGGTATAGTACTTTCTCTAATGA	1
EBOV-400_59_LEFT	GGGACATGGCAAGCTGAGTTTA	1
EBOV-400_59_RIGHT	TCACACATGCTGCATTGTGAAT	1
EBOV-400_61_LEFT	CGAAAGGTCTGGGCTCATATTGT	1
EBOV-400_61_RIGHT	CCAGGAATCCTTTTTGCAACGT	1
EBOV-400_02_LEFT	TCACAGTCAAACAAGCAAGATTGA	2
EBOV-400_02_RIGHT	CTCCCTGGTACGCATGATGAAG	2
EBOV-400_04_LEFT	GTAGGAGAAAAGGCTTGCCTTGA	2
EBOV-400_04_RIGHT	CATACTCTCCATGCTTGGCCAG	2
EBOV-400_06_LEFT	ACCATCTTGGACTTGATGATCAGG	2
EBOV-400_06_RIGHT	ACTGGCTTAGTGTCCTCATCGT	2
EBOV-400_08_LEFT	ATCAACGAAGAGGCAGACCCA	2
EBOV-400_08_RIGHT	TTTTTCAGTGAGCCATGGTGGA	2
EBOV-400_10_LEFT	ACGAAACCAGGAAGAATTTTTGATGT	2
EBOV-400_10_RIGHT	TGCCCTTTGTTCTGGTTGTCAT	2
EBOV-400_12_LEFT	AGGAGGTAGTGCAAACATTAGCT	2
EBOV-400_12_RIGHT	TTGCCGAAATGTCAGGTTTCCC	2
EBOV-400_14_LEFT	ATCCGCTCTCGAGGTGACATTC	2
EBOV-400_14_RIGHT	CTCTCAGTCGAGGTAGGTTTTTCT	2
EBOV-400_16_LEFT	ATGACACCATCGACCATGCCA	2
EBOV-400_16_RIGHT	TCATCGGTCCATGTTGCAGC	2
EBOV-400_18_LEFT	AGATCGTTCCAATTGATCCAACCA	2
EBOV-400_18_RIGHT	TGAGTCTTTTGTTAAGTTCTACATTTAGGA	2
EBOV-400_20_LEFT	GGTTGTTCACCATCCAAGTACAGA	2
EBOV-400_20_RIGHT	AGTCCAACTGATCTCAATTGATTTGT	2
EBOV-400_22_LEFT	TGCACAAAGTATCAGGAACGGG	2
EBOV-400_22_RIGHT	AGTTTTCCCGTGGTATTGCTCC	2
EBOV-400_24_LEFT	GCAATGGTTCAAGTGCACAGTC	2
EBOV-400_24_RIGHT	GGTAATTAAGCCTAGCTTCCCGC	2
EBOV-400_26_LEFT	TGGCCAACGAGACGACTCAA	2
EBOV-400_26_RIGHT	TGATTTGAGGCTGAACTTTGCC	2
EBOV-400_28_LEFT	CCTTGATTCTACAATCATGACAGTTGT	2
EBOV-400_28_RIGHT	TTTTTCAAGGTCGGACATATGTCTTT	2
EBOV-400_30_LEFT	GGCGAGACAAGACATCAGAACC	2
EBOV-400_30_RIGHT	CCCTCATCAGACCATGAGCATG	2
EBOV-400_32_LEFT	TCGCTCGTAATATAACCTGCCA	2
EBOV-400_32_RIGHT	GGACTACCCTGGAATAGTACTTTGC	2
EBOV-400_34_LEFT	GAACATCACTTTGAGCGCCCTC	2
EBOV-400_34_RIGHT	ACAGCGGTGATTCAATTGTGGA	2
EBOV-400_36_LEFT	ACATGTCGTGAACTACAACGGA	2
EBOV-400_36_RIGHT	GCTGGCTTACGGTGAGGATTTA	2
EBOV-400_38_LEFT	CATGTGTTAGGTTTCACAGATTCTATATATTAC	2
EBOV-400_38_RIGHT	CAGTGCCTTGAGAAGAATTGGGA	2
EBOV-400_40_LEFT	CGAAGGGGTAGATTAAACCGAGG	2
EBOV-400_40_RIGHT	ACCCATCAGACCCTAATATGGAGA	2
EBOV-400_42_LEFT	TTCAAAAACACTGGGGGCATCC	2
EBOV-400_42_RIGHT	TAGGCTCGAATACTGCATCCCA	2
EBOV-400_44_LEFT	ACTCGCAATGTTCAAACACTTTGT	2
EBOV-400_44_RIGHT	TCCCTCCCATATGACCCCTGTA	2
EBOV-400_46_LEFT	TGCCTGCGGAATCTTTTTAAAACC	2
EBOV-400_46_RIGHT	AAACATTTCTCAGGATTCAAGAAGGAT	2
EBOV-400_48_LEFT	ACCCTAAGTGCGAAAAACAAACT	2
EBOV-400_48_RIGHT	GGCTCCAATAAGAGGTCTTCCC	2
EBOV-400_50_LEFT	AATGTCCTTCCGCAGCCTTAAG	2
EBOV-400_50_RIGHT	TTGGATATCTGTAGCCTCAGTGTT	2
EBOV-400_52_LEFT	TATCTGGATGGGAGCTAGCCAA	2
EBOV-400_52_RIGHT	TAATCTGGCCGCATCTGAGAGT	2
EBOV-400_54_LEFT	TGTTTACACATGTAAGAGTACAGCCA	2
EBOV-400_54_RIGHT	ACGGTGTGAGATTATTTGATGACCT	2
EBOV-400_56_LEFT	TGACACTAGCAGAGGGAGAAGG	2
EBOV-400_56_RIGHT	CTTAGAAAGACTTTAAGGACCACTGC	2
EBOV-400_58_LEFT	TGGCTAAGTCATTTAACTCAGTATGC	2
EBOV-400_58_RIGHT	ACGTTCTTCACAATTGCAATCTCT	2
EBOV-400_60_LEFT	TCCGGATGGTCTCTACAGGTTT	2
EBOV-400_60_RIGHT	TTTGTTTTAAGAAGTCGTGTTATCAACC	2

Supplemental Table 2: Bayesian Model Fitting Estimates

**Clock Model, 2020 Mbandaka Sequences**	**Loglikelihood Path Sampling**		**Loglikelihood Stepping Stone**	
	Average	Standard Deviation	Average	Standard Deviation
Fixed Local Clock, Skygrid	-29192.6	1.0	-29192.5	1.2
Relaxed Clock (UCLN), constant population	-29186.4	0.6	-29186.6	0.8
Relaxed Clock (UCLN) - Skygrid	-29179.5	1.1	-29179.7	1.2
Strict Clock (UCLN) - Skygrid	-29192.1	0.7	-29192.3	0.9
Relaxed Clock (UCLN), exponential population	-29182.9	0.9	-29182.9	0.9

Bayes Factor - Path Sampling

	FLC, Skygrid	Strict Clock, Skygrid	UCLN, constant	UCLN, Skygrid	UCLN, exponential
Fixed Local Clock, Skygrid		0.26	5.96	**12.87**	9.67
Relaxed Clock (UCLN), constant population	-0.26		5.70	**12.61**	9.41
Relaxed Clock (UCLN) - Skygrid	-5.96	-5.70		**6.91**	3.71
Strict Clock (UCLN) - Skygrid	-12.87	-12.61	-6.91		-3.20
Relaxed Clock (UCLN), exponential population	-9.67	-9.41	-3.71	**3.20**	

Bayes Factor - Stepping Stone

	FLC, Skygrid	Strict Clock, Skygrid	UCLN, constant	UCLN, Skygrid	UCLN, exponential
Fixed Local Clock, Skygrid		0.39	6.07	**13.02**	9.60
Relaxed Clock (UCLN), constant population	-0.39		5.68	**12.63**	9.21
Relaxed Clock (UCLN) - Skygrid	-6.07	-5.68		**6.95**	3.52
Strict Clock (UCLN) - Skygrid	-13.02	-12.63	-6.95		-3.42
Relaxed Clock (UCLN), exponential population	-9.60	9.21	-3.52	**3.42**	

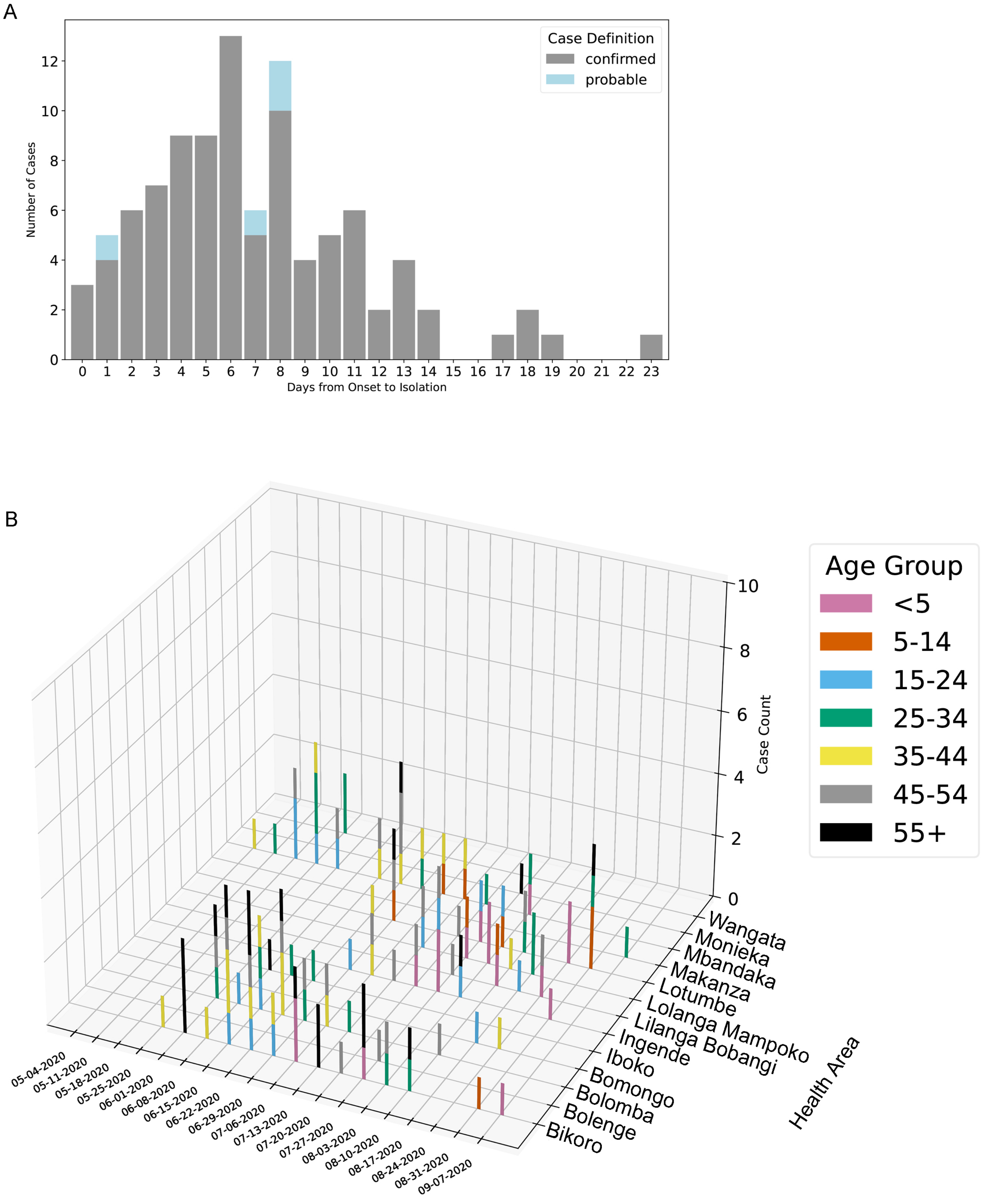

Supplemental Figure 1: Epidemiological characteristics of EBOV spread in the Equateur district.

Supplemental Table 3: Summary of Sequencing Results and Metadata

Genbank Accession Number	EPI ID	*Zaire Ebolavirus* variant	ID Labo	Sampling Type	Age	Type of age	Sex	Province	Health zone	Onset Date	Sampling Date	CT Xpert GP	CT Xpert NP	Coverage	NTC Coverage	Preparation Date	Sequencing Date	Platform	Pipeline
OR084849	RDCEQT000008	Mbandaka	MBK-0003-20	1st sampling	31	year	Female	Equator	Mbandaka	2020-05-26	2020-05-31	26.4	21.6	99.9%	0.0%	2021-09-14	2021-09-22	Illumina	EBOV_assemble_genomes.sh
OR084850	RDCEQT000010	Mbandaka	MBK-0019-20	2nd sampling	31	year	Female	Equator	Mbandaka	2020-05-28	2020-05-31	24.5	20.3	99.9%	0.0%	2021-09-14	2021-09-23	Illumina	EBOV_assemble_genomes.sh
OR084851	RDCEQT000016	Mbandaka	MBK-0001-20					Equateur	Mbandaka	2020-05-19				99.9%	0.0%	2021-09-14	2021-09-23	Illumina	EBOV_assemble_genomes.sh
OR084852	RDCEQT000017	Mbandaka	MBK-0018-20	1st sampling	32	year	Male	Equator	Wangata	2020-05-27	2020-06-02	24.3	18.6	99.9%	0.0%	2021-09-14	2021-09-23	Illumina	EBOV_assemble_genomes.sh
OR084853	RDCEQT000018	Mbandaka	MBK-0021-20	1st sampling	38	year	Male	Equator	Mbandaka	2020-05-26	2020-06-03	28.1	24.7	99.9%	0.0%	2021-09-14	2021-09-22	Illumina	EBOV_assemble_genomes.sh
OR084854	RDCEQT000019	Mbandaka	MBK-0024-20	1st sampling	33	year	Female	Equator	Wangata	2020-05-26		23.7	19.1	96.6%	0.0%	2020-09-01	2020-09-02	Minion	Artic
OR084848	RDCEQT000029	Tumba	BIK-0014-20	1st sampling	55	year	Female	Equator	Iboko	2020-06-15	2020-06-18	39.8	37.7	75.7%		2020-07-16	2020-07-20	Illumina	iVAR
OR084855	RDCEQT000042	Mbandaka	BIK-0007-20	1st sampling	45	year	Male	Equator	Bikoro	2020-06-07	2020-06-17	28.7	23.1	96.5%	0.0%	2020-09-01	2020-09-04	Minion	Artic
OR084856	RDCEQT000046	Mbandaka	BIK-0001-20	1st sampling	59	year	Female	Equator	Bikoro	2020-06-07	2020-06-10	24.8	20.6	98.0%	0.0%	2020-09-01	2020-09-04	Minion	Artic
OR084857	RDCEQT000074	Mbandaka	MBK-0043-20	1st sampling	22	year	Female	Equator	Mbandaka	2020-05-31	2020-06-06	24.3	20	99.9%	0.0%	2021-09-14	2021-09-23	Illumina	EBOV_assemble_genomes.sh
OR084846	RDCEQT000151	Tumba	MBK-0067-20	1st sampling	19	year	Male	Equator	Iboko	2020-06-04	2020-06-12	35.1	32.4	96.3%	26.8%	2020-07-01	2020-07-07	Illumina	iVAR
OR084847	RDCEQT000199	Tumba		1st sampling	30	year	Male	Equator	Iboko	2020-06-16	2020-06-18	35.6	34.6	83.8%		2020-07-16	2020-07-20	Illumina	iVAR
OR084858	RDCEQT000227	Mbandaka	MBK-0092-20	1st sampling	34	year	Male	Equator	Bolomba	2020-06-05	2020-06-15	34.3	30.2	92.3%	0.0%	2020-09-01	2020-09-04	Minion	Artic
OR084859	RDCEQT000299	Mbandaka	MBK-0134-20	2nd sampling	46	year	Male	Equator	Bolomba	2020-05-28	2020-06-20	30.2	25.8	99.8%	0.0%	2021-09-14	2021-11-19	Illumina	EBOV_assemble_genomes.sh
OR084928	RDCEQT000300	Mbandaka	MBK-0110-20					Equateur	Bolomba	2020-06-06				99.8%	0.0%	2021-09-14	2021-11-19	Illumina	EBOV_assemble_genomes.sh
OR084860	RDCEQT000301	Mbandaka	MBK-0096-20	1st sampling	17	year	Female	Equator	Bolomba	2020-06-08	2020-06-16	36.6	33.3	91.0%	0.0%	2020-09-01	2020-09-04	Minion	Artic
OR084861	RDCEQT000456	Mbandaka	BIK-0008-20	1st sampling	43	year	Female	Equator	Bikoro	2020-06-15		28.2	22.5	97.6%	0.0%	2020-09-01	2020-09-04	Minion	Artic
OR084862	RDCEQT000458	Mbandaka	BIK-0019-20	1st sampling	35	year	Male	Equator	Bikoro	2020-06-17	2020-06-21	26.1	21.1	96.5%	0.0%	2020-09-01	2020-09-02	Minion	Artic
OR084863	RDCEQT000474	Mbandaka	BIK-0076-20	1st sampling	24	year	Female	Equator	Bikoro	2020-06-22	2020-06-29	24.6	19.1	92.3%	0.0%	2020-09-01	2020-09-04	Minion	Artic
OR084864	RDCEQT000492	Mbandaka	BIK-0118-20	1st sampling	80	year	Male	Equator	Bikoro	2020-06-22	2020-07-03	28.4	22.4	93.4%	0.0%	2020-09-01	2020-09-04	Minion	Artic
OR084865	RDCEQT000514	Mbandaka	BIK-0039-20	1st sampling	45	year	Female	Equator	Bikoro	2020-06-22	2020-06-24	30.6	25.7	94.9%	0.0%	2020-09-01	2020-09-04	Minion	Artic
OR084866	RDCEQT000571	Mbandaka	MBK-0138-20					Equateur	Wangata	2020-06-17				99.9%	0.0%	2021-09-14	2021-11-19	Illumina	EBOV_assemble_genomes.sh
OR084867	RDCEQT000616	Mbandaka	BIK-0072-20	1st sampling	35	year	Male	Equator	Bikoro	2020-06-21	2020-06-28	31.8	26.6	92.3%	0.0%	2020-09-01	2020-09-04	Minion	Artic
OR084868	RDCEQT000617	Mbandaka	BIK-0074-20	1st sampling	19	year	Male	Equator	Bikoro	2020-06-26	2020-06-29	28.8	23.2	94.9%	0.0%	2020-09-01	2020-09-04	Minion	Artic
OR084869	RDCEQT000796	Mbandaka	MBK-0168-20	1st sampling	35	year	Female	Equator	Mbandaka	2020-06-18	2020-06-25	30.2	25.6	98.9%	0.0%	2021-09-14	2021-09-22	Illumina	EBOV_assemble_genomes.sh
OR084870	RDCEQT000869	Mbandaka	MBK-0255-20	1st sampling	48	year	Male	Equator	Mbandaka	2020-06-23	2020-06-30	28.5	24.7	87.8%	0.0%	2020-09-01	2020-09-04	Minion	Artic
OR084871	RDCEQT002101	Mbandaka	MBK-0859-20	1st sampling	49	year	Male	Equator	Bolomba	2020-07-11		37.3	33.6	90.7%	0.0%	2020-09-01	2020-09-02	Minion	Artic
OR084872	RDCEQT002103	Mbandaka	MBK-0953-20	1st sampling	50	year	Male	Equator	Ingende	2020-07-10		29.3	26.5	98.0%	0.0%	2020-09-01	2020-09-02	Minion	Artic
OR084873	RDCEQT002106	Mbandaka	MBK-0727-20	1st sampling	37	year	Male	Equator	Mbandaka	2020-07-06	2020-07-13	24.6	19.6	98.0%	0.0%	2020-09-01	2020-09-02	Minion	Artic
OR084874	RDCEQT002107	Mbandaka	MBK-0908-20	1st sampling	60	year	Male	Equator	Lotumbe	2020-07-02	2020-07-12	30.1	24.8	96.6%	0.0%	2020-09-01	2020-09-02	Minion	Artic
OR084875	RDCEQT002111	Mbandaka	BIK-0294-20	1st sampling	58	year	Female	Equator	Bikoro	2020-07-12	2020-07-14	32.1	27	98.0%	0.0%	2020-09-01	2020-09-02	Minion	Artic
OR084876	RDCEQT002112	Mbandaka	MBK-0829-20	1st sampling	48	year	Male	Equator	Bolomba	2020-07-05		38.2	32.3	90.7%	0.0%	2020-09-01	2020-09-02	Minion	Artic
OR084877	RDCEQT002113	Mbandaka	BIK-0183-20					Equateur	Bikoro	2020-07-01				74.1%	0.0%	2021-09-14	2021-11-19	Illumina	EBOV_assemble_genomes.sh
OR084878	RDCEQT002117	Mbandaka	BIK-0446-20	1st sampling	4	year	Female	Equator	Bikoro	2020-07-13	2020-07-23	32.1	26.8	98.0%	0.0%	2020-10-08	2020-10-15	Minion	Artic
OR084879	RDCEQT002118	Mbandaka	BIK-0425-20	1st sampling	32	year	Male	Equator	Bikoro	2020-07-16	2020-07-21	26.6	21	80.0%	0.0%	2020-10-08	2020-10-15	Minion	Artic
OR084880	RDCEQT002119	Mbandaka	MBK-1147-20	1st sampling	35	year	Male	Equator	Ingende	2020-07-12	2020-07-20	27.9	25	92.0%	0.0%	2020-09-01	2020-09-02	Minion	Artic
OR084881	RDCEQT002121	Mbandaka	MBK-1204-20	1st sampling	30	year	Male	Equator	Bolomba	2020-07-14	2020-07-20	41.7	35.2	89.3%	0.0%	2020-09-01	2020-09-02	Minion	Artic
OR084882	RDCEQT002122	Mbandaka	BIK-0575-20		59	year	Male	Equator	Bikoro	2020-07-18	2020-07-27	29.7	24.5	98.0%	0.0%	2020-10-08	2020-10-15	Minion	Artic
OR084883	RDCEQT002123	Mbandaka	BLB-0070-20		37	year	Female	Equator	Bolomba	2020-07-11			32.1	93.7%	0.0%	2020-09-01	2020-09-02	Minion	Artic
OR084884	RDCEQT002124	Mbandaka	MBK-1498-20	1st sampling	6	year	Male	Equator	Ingende	2020-07-25	2020-07-28	30.3	27.3	91.2%	0.0%	2021-09-14	2021-09-22	Illumina	EBOV_assemble_genomes.sh
OR084885	RDCEQT002125	Mbandaka	MBK-1488-20	1st sampling	46	year	Male	Equator	Lotumbe	2020-07-21		35.2	30.2	98.5%	0.0%	2021-09-14	2021-11-12	Illumina	EBOV_assemble_genomes.sh
OR084886	RDCEQT002127	Mbandaka	MBK-1599-20	1st sampling	47	year	Female	Equator	Ingende	2020-07-18		36.2	33.2	89.8%	0.0%	2020-09-01	2020-09-02	Minion	Artic
OR084887	RDCEQT002128	Mbandaka	MBK-1807-20	1st sampling	47	year	Female	Equator	Lilanga Bobangi	2020-08-02		35.4	32.7	92.0%	0.0%	2020-09-01	2020-09-02	Minion	Artic
OR084888	RDCEQT002129	Mbandaka	MBK-1658-20	1st sampling	28	year	Male	Equator	Ingende	2020-08-01		32.4	30.1	99.9%	0.0%	2021-09-14	2021-09-23	Illumina	EBOV_assemble_genomes.sh
OR084889	RDCEQT002130	Mbandaka	MBK-1662-20	1st sampling	2	year	Female	Equator	Ingende	2020-08-01		33.8	30.7	96.5%	0.0%	2020-09-01	2020-09-02	Minion	Artic
OR084890	RDCEQT002132	Mbandaka	MBK-1715-20	1st sampling	2	year	Male	Equator	Ingende	2020-08-01		34.9	31.7	84.8%	0.0%	2020-09-01	2020-09-02	Minion	Artic
OR084891	RDCEQT002134	Mbandaka	MBK-1818-20	1st sampling	45	year	Male	Equator	Ingende	2020-08-01		36.6	34.1	88.5%	0.0%	2020-09-01	2020-09-02	Minion	Artic
OR084892	RDCEQT002135	Mbandaka	BIK-1168-20		65	year	Male	Equator	Bikoro	2020-08-03		28.2	23.1	93.5%	0.0%	2020-09-01	2020-09-02	Minion	Artic
OR084893	RDCEQT002136	Mbandaka	BIK-1128-20		7	month	Female	Equator	Bikoro	2020-08-02		33.4	28.1	90.7%	0.0%	2020-09-01	2020-09-02	Minion	Artic
OR084894	RDCEQT002137	Mbandaka	BIK-1138-20		61	year	Female	Equator	Bikoro	2020-08-03				93.5%	0.0%	2020-09-01	2020-09-02	Minion	Artic
OR084895	RDCEQT002138	Mbandaka	MBK-1969-20		7	year	Female	Equator	Lilanga Bobangi	2020-08-09		31.3	25	94.9%	0.0%	2020-09-01	2020-09-02	Minion	Artic
OR084896	RDCEQT002139	Mbandaka	MBK-1958-20		3	month	Female	Equator	Lolanga Mampoko	2020-08-02		31.4	24.8	98.0%	0.0%	2020-09-01	2020-09-02	Minion	Artic
OR084897	RDCEQT002140	Mbandaka	BIK-1434-20		26	year	Male	Equator	Bikoro	2020-08-05	2020-08-15	28.4	23.2	99.4%	0.0%	2020-09-03	2020-09-05	Minion	Artic
OR084898	RDCEQT002141	Mbandaka	MBK-2055-20					Equateur	Lotumbe	2020-08-01				99.9%	0.0%	2021-09-14	2021-09-22	Illumina	EBOV_assemble_genomes.sh
OR084931	RDCEQT002143	Mbandaka	MBK-2247-20	2nd sampling	8	year	Female	Equator	Lotumbe	2020-08-09	2020-08-19	35.6	31.8	97.8%	20.6%	2020-09-11	2020-09-11	Minion	Artic
OR084899	RDCEQT002144	Mbandaka	MBK-2080-20		8	year	Male	Equator	Lolanga Mampoko	2020-08-05	2020-08-12	29.3	24.5	99.4%	0.0%	2020-09-03	2020-09-05	Minion	Artic
OR084900	RDCEQT002145	Mbandaka	MBK-2081-20		6	year	Male	Equator	Lolanga Mampoko	2020-08-05	2020-08-12	30.9	26.6	96.0%	0.0%	2020-09-03	2020-09-05	Minion	Artic
OR084901	RDCEQT002146	Mbandaka	MBK-2021-20		1	year	Female	Equator	Mbandaka	2020-08-04		24.2	21.4	96.6%	0.0%	2020-09-01	2020-09-02	Minion	Artic
OR084929	RDCEQT002148	Mbandaka	MBK-2099-20					Equateur	Lotumbe	2020-08-04				99.9%	0.0%	2021-09-14	2021-11-12	Illumina	EBOV_assemble_genomes.sh
OR084902	RDCEQT002149	Mbandaka	MBK-2154-20		38	year	Male	Equator	Lolanga Mampoko	2020-08-10		28.3	24.2	99.4%	0.0%	2020-09-03	2020-09-05	Minion	Artic
OR084903	RDCEQT002151	Mbandaka	MBK-2190-20		50	year	Male	Equator	Bolenge	2020-08-02	2020-08-13	38.9	30.5	96.4%	0.0%	2020-09-03	2020-09-05	Minion	Artic
OR084904	RDCEQT002152	Mbandaka	MBK-2171-20		28	year	Female	Equator	Lotumbe	2020-08-16	2020-08-16	34.4	29.1	98.0%	0.0%	2020-09-03	2020-09-05	Minion	Artic
OR084905	RDCEQT002153	Mbandaka	BIK-1631-20		51	year	Female	Equator	Bikoro	2020-08-09	2020-08-21	34.4	29.4	87.9%	0.0%	2020-09-03	2020-09-05	Minion	Artic
OR084906	RDCEQT002154	Mbandaka	MBK-2308-20		15	year	Female	Equator	Lilanga Bobangi	2020-08-21	2020-08-22	33.5	29.5	93.6%	0.0%	2020-09-03	2020-09-05	Minion	Artic
OR084907	RDCEQT002155	Mbandaka	MBK-2307-20		4	year	Female	Equator	Lilanga Bobangi	2020-08-15	2020-08-21	33.6	29.7	93.6%	0.0%	2020-09-03	2020-09-05	Minion	Artic
OR084908	RDCEQT002156	Mbandaka	MBK-2135-20		24	year	Male	Equator	Ingende	2020-08-10	2020-08-16	38.4	34.9	86.5%	0.0%	2020-09-03	2020-09-05	Minion	Artic
OR084909	RDCEQT002159	Mbandaka	BIK-1765-20		32	year	Male	Equator	Bikoro	2020-08-13	2020-08-25	26.2	20.5	96.1%	0.0%	2020-09-03	2020-09-05	Minion	Artic
OR084910	RDCEQT002163	Mbandaka	MBK-2551-20	1st sampling				Equator	Bomongo	2020-08-21		31	26.8	99.9%	0.0%	2021-09-14	2021-11-12	Illumina	EBOV_assemble_genomes.sh
OR084911	RDCEQT002164	Mbandaka	MBK-2594-20	1st sampling	4	year	Female	Equator	Lotumbe	2020-08-31	2020-09-03	38.2	34.2	99.4%	0.0%	2020-10-08	2020-10-15	Minion	Artic
OR084926	RDCEQT002168	Mbandaka	MBK-2627-20	1st sampling	38	year	Male	Equator	Bomongo	2020-08-28	2020-09-03	34	30.3	96.7%	0.0%	2020-09-17	2020-09-18	Minion	Artic
OR084925	RDCEQT002169	Mbandaka	MBK-2620-20	1st sampling	34	year	Female	Equator	Lolanga mampoko	2020-08-20	2020-08-23	34.1	30.4	92.4%	0.0%	2020-09-17	2020-09-18	Minion	Artic
OR084924	RDCEQT002170	Mbandaka	MBK-2609-20	1st sampling	34	year	Female	Equator	Lolanga mampoko	2020-08-20		34.9	31.3	92.7%	0.0%	2020-09-17	2020-09-18	Minion	Artic
OR084927	RDCEQT002171	Mbandaka	MBK-2585-20	1st sampling	78	year	Male	Equator	Lotumbe	2020-09-01	2020-09-06	35.8	32.5	99.4%	0.0%	2020-10-08	2020-10-15	Minion	Artic
OR084912	RDCEQT002501	Mbandaka	MBK-0977-20	1st sampling	38	year	Male	Equator	Mbandaka	2020-06-07	2020-07-19	28.7	24.8	93.7%	0.0%	2020-09-01	2020-09-02	Minion	Artic
OR084913	RDCEQT002502	Mbandaka	MBK-0984-20	1st sampling	11	year	Female	Equator	mbandaka	2020-07-09		30.5	27.1	93.7%	0.0%	2020-09-01	2020-09-02	Minion	Artic
OR084914	RDCEQT002614	Mbandaka	MBK-1243-20	1st sampling	7	year	Male	Equator	Mbandaka	2020-07-20	2020-07-22	28.4	24.8	99.4%	0.0%	2020-09-01	2020-09-02	Minion	Artic
OR084915	RDCEQT003001	Mbandaka	MBK-0396-20	1st sampling	51	year	Male	Equator	Mbandaka	2020-06-28		26.7	23.3	99.9%	0.0%	2021-09-14	2021-09-23	Illumina	EBOV_assemble_genomes.sh
OR084916	RDCEQT003006	Mbandaka	BIK-0102-20	1st sampling	65	year	Male	Equator	Bikoro	2020-06-26	2020-07-02	24.9	19.4	94.9%	0.0%	2020-09-01	2020-09-04	Minion	Artic
OR084930	RDCEQT003007	Mbandaka	MBK-2175-20					Equateur	Lotumbe	2020-07-27				98.8%	0.0%	2021-09-14	2021-09-27	Illumina	EBOV_assemble_genomes.sh
OR084917	RDCEQT003008	Mbandaka	MBK-0454-20	1st sampling	85	year	Male	Equator	Mbandaka	2020-06-23	2020-07-06	17.5	13.5	76.5%	0.0%	2020-09-01	2020-09-04	Minion	Artic
OR084918	RDCEQT003011	Mbandaka	BIK-0042-20	1st sampling	46	year	Male	Equator	bikoro	2020-06-23		28.9	23.5	94.9%	0.0%	2020-09-01	2020-09-04	Minion	Artic
OR084919	RDCEQT003013	Mbandaka	MBK-0221-20	2nd sampling	40	year	Female	Equator	bolomba	2020-06-19	2020-06-26	33.5	28.1	97.7%	0.0%	2021-09-14	2021-09-22	Illumina	EBOV_assemble_genomes.sh
OR084920	RDCEQT003014	Mbandaka	MBK-0228-20	1st sampling	25	year	Female	Equator	Bolomba	2020-06-17	2020-06-26	35.3	30.4	99.7%	0.0%	2021-09-14	2021-09-22	Illumina	EBOV_assemble_genomes.sh
OR084921	RDCEQT003018	Mbandaka	MBK-0299-20	1st sampling	35	year	Female	Equator	Mbandaka	2020-06-26	2020-07-01	23.9	21.3	99.9%	0.0%	2021-09-14	2021-09-23	Illumina	EBOV_assemble_genomes.sh
OR084922	RDCEQT003019	Mbandaka	BIK-0094-20	1st sampling	60	year	Male	Equator	Bikoro	2020-06-29		26.3	20.7	82.0%	0.0%	2020-09-01	2020-09-04	Minion	Artic
OR084923	RDCEQT003256	Mbandaka	MBK-2001-20		56	year	Male	Equator	Monieka	2020-08-03		31.1	27.1	96.5%	0.0%	2020-09-01	2020-09-02	Minion	Artic
OR084932	RDCEQT009903	Mbandaka	MBK-3154-20		34	year	Female	Equator	Makanza	2020-09-16		29.5	25.7	79.8%	0.0%	2020-10-08	2020-10-15	Minion	Artic

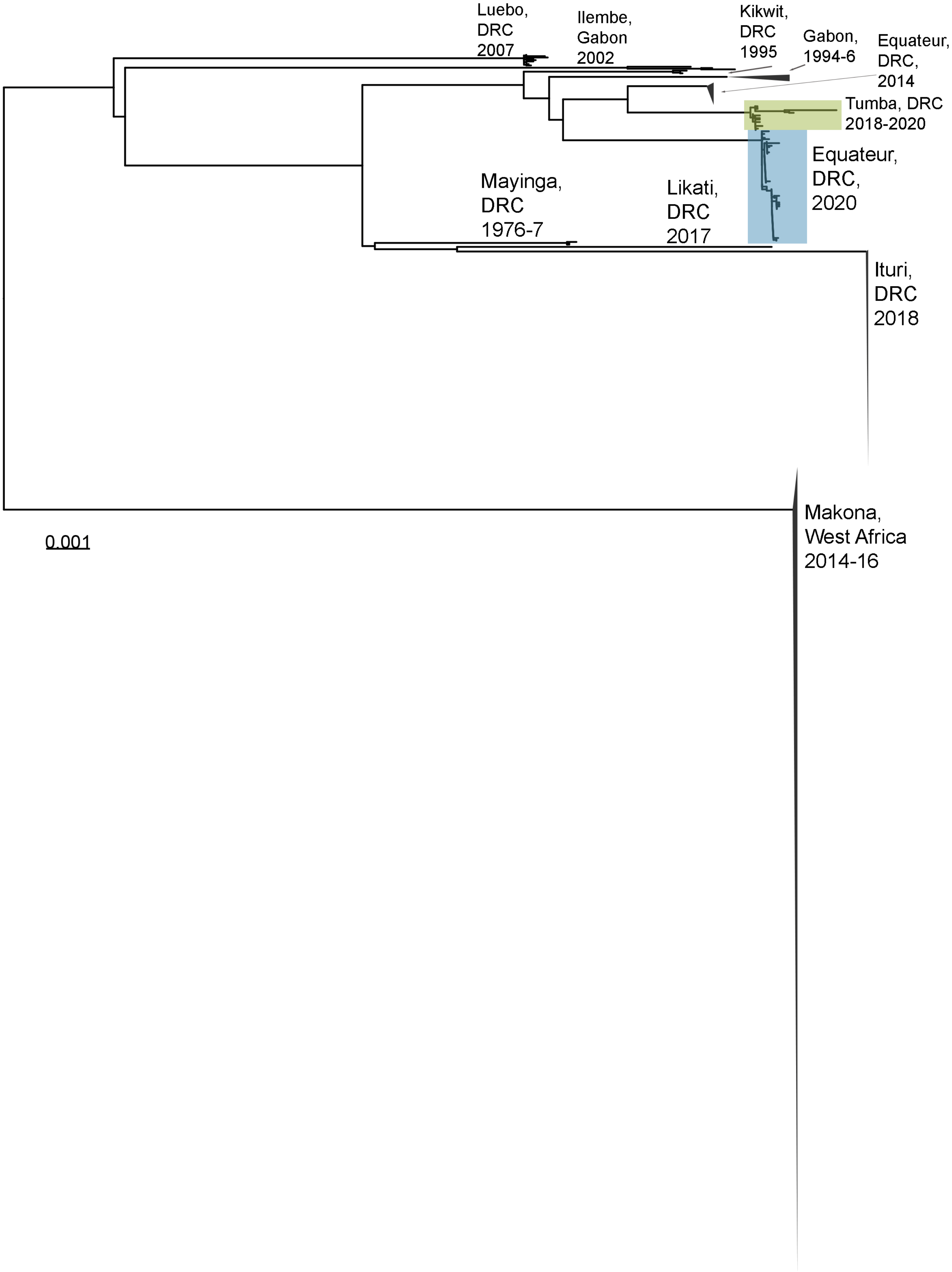

Supplementary Figure 2: Inferred evolutionary relationships for the *Orthoebolavirus zairense* species

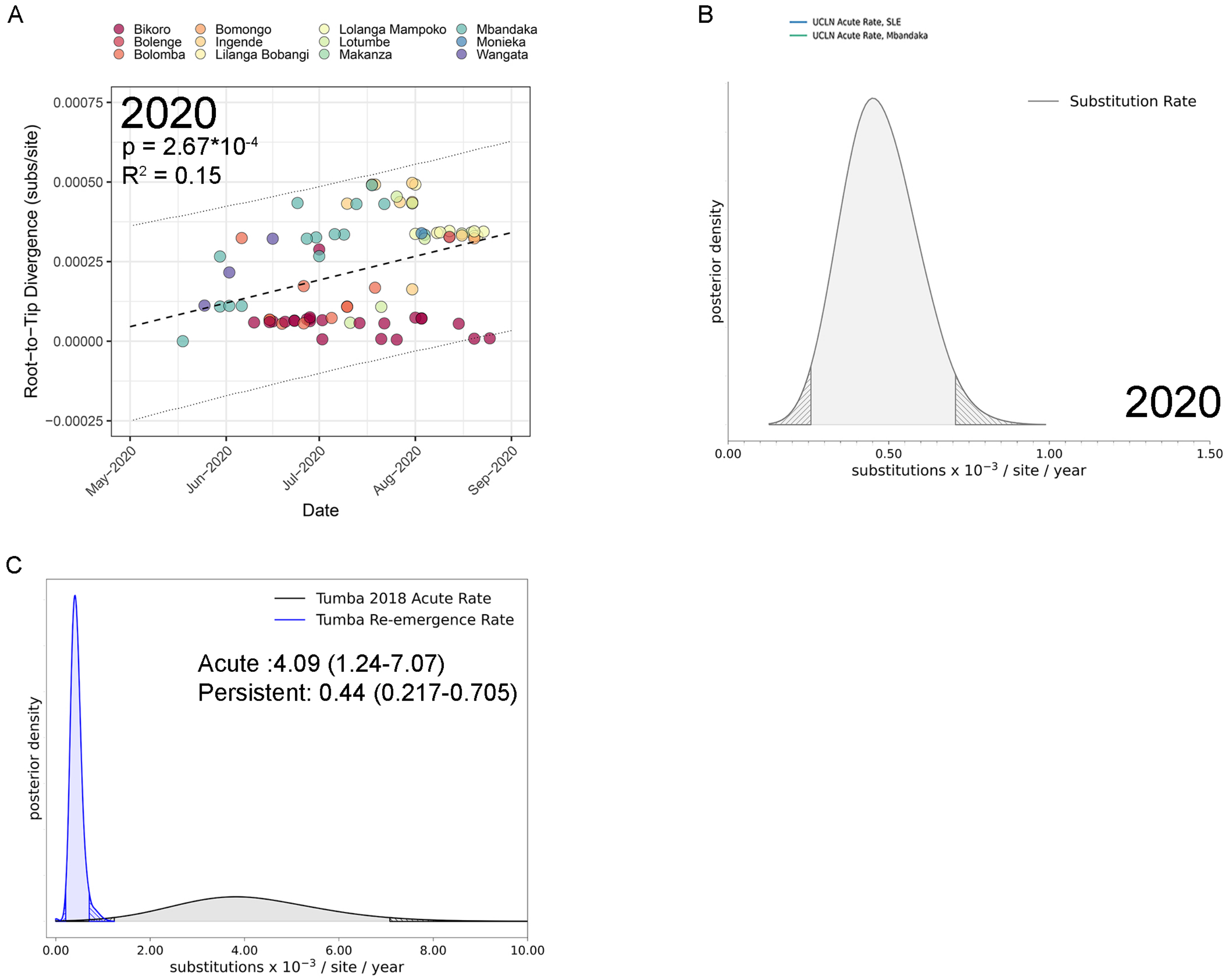

Supplemental Figure 3: Inferred phylogenetic relationships from the Equateur_2020 EBOV outbreak.

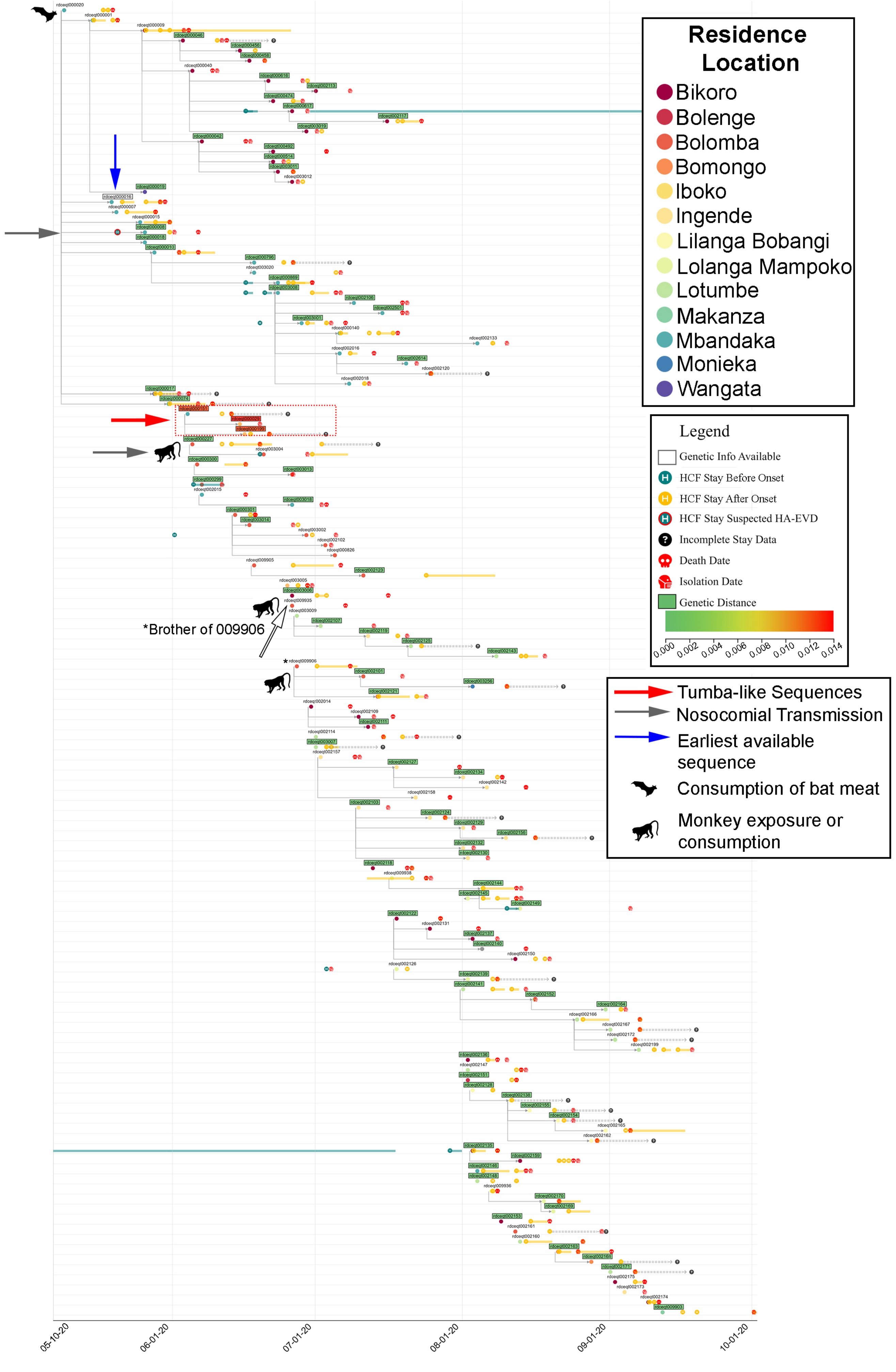

Supplemental Figure 4: Overview of Equateur 2020 EBOV outbreak, combined epidemiological and genetic data.
